# Harnessing Mechanistic Knowledge on Beneficial Versus Deleterious IFN-I Effects to Design Innovative Immunotherapies Targeting Cytokine Activity to Specific Cell Types

**DOI:** 10.3389/fimmu.2014.00526

**Published:** 2014-10-30

**Authors:** Elena Tomasello, Emeline Pollet, Thien-Phong Vu Manh, Gilles Uzé, Marc Dalod

**Affiliations:** ^1^UM2, Centre d’Immunologie de Marseille-Luminy (CIML), Aix-Marseille University, Marseille, France; ^2^U1104, Institut National de la Santé et de la Recherche Médicale (INSERM), Marseille, France; ^3^UMR7280, Centre National de la Recherche Scientifique (CNRS), Marseille, France; ^4^UMR 5235, Centre National de la Recherche Scientifique (CNRS), University Montpellier II, Montpellier, France

**Keywords:** type I interferons, dendritic cells, chronic viral infections, immunotherapy, bioengineering

## Abstract

Type I interferons (IFN-I) were identified over 50 years ago as cytokines critical for host defense against viral infections. IFN-I promote anti-viral defense through two main mechanisms. First, IFN-I directly reinforce or induce *de novo* in potentially all cells the expression of effector molecules of intrinsic anti-viral immunity. Second, IFN-I orchestrate innate and adaptive anti-viral immunity. However, IFN-I responses can be deleterious for the host in a number of circumstances, including secondary bacterial or fungal infections, several autoimmune diseases, and, paradoxically, certain chronic viral infections. We will review the proposed nature of protective versus deleterious IFN-I responses in selected diseases. Emphasis will be put on the potentially deleterious functions of IFN-I in human immunodeficiency virus type 1 (HIV-1) infection, and on the respective roles of IFN-I and IFN-III in promoting resolution of hepatitis C virus (HCV) infection. We will then discuss how the balance between beneficial versus deleterious IFN-I responses is modulated by several key parameters including (i) the subtypes and dose of IFN-I produced, (ii) the cell types affected by IFN-I, and (iii) the source and timing of IFN-I production. Finally, we will speculate how integration of this knowledge combined with advanced biochemical manipulation of the activity of the cytokines should allow designing innovative immunotherapeutic treatments in patients. Specifically, we will discuss how induction or blockade of specific IFN-I responses in targeted cell types could promote the beneficial functions of IFN-I and/or dampen their deleterious effects, in a manner adapted to each disease.

## Introduction

Type I interferons (IFN-I) were the first cytokines discovered, over 50 years ago, based on their potent anti-viral effects (1, 2). IFN-I play a crucial, non-redundant role in vertebrate anti-viral defenses (3–5). IFN-I also mediate protective effects in other physiopathological contexts, including cancer (6) and multiple sclerosis (MS) (7). On the contrary, IFN-I responses can be deleterious in a number of circumstances, including bacterial or fungal infections (8–10), many autoimmune diseases (11), and, paradoxically, certain chronic viral infections (12–14). It is only recently that an integrated picture has emerged of the cellular and molecular mechanisms regulating the production of IFN-I and underlying their functions. Much knowledge was gained recently on another class of potent innate anti-viral interferons, the lambda, or type III IFNs (IFN-III). We will review knowledge on IFN-I/III (IFNs) and discuss how it could be harnessed to develop innovative therapeutic strategies aimed at surgically tuning IFN activity toward protective responses in a manner adapted to each disease. We will focus on IFN-α/β/λ because they are the best characterized IFNs and already used therapeutically. Recent reviews are covering information on other IFN-I subsets including IFN-ε, which is produced at mucosal sites and promotes local anti-viral defenses (15, 16).

Dendritic cells (DCs) are rare heterogeneous mononuclear phagocytes functionally characterized by their unique efficacy for antigen-specific activation of naïve T lymphocytes. DCs are sentinel cells of the immune system, able to sense and integrate a variety of danger input signals for delivery of output signals instructing the activation and functional polarization of effector immune cells. In mammals, five major DC subsets exist, which differ in their expression of innate immune recognition receptors (I_2_R_2_s) and in their functional specialization: monocyte-derived DCs (MoDCs), Langerhans cells, CD11b^+^ DCs, XCR1^+^ DCs, and plasmacytoid DCs (pDCs) (17). A recurrent theme of this review will be the intricate relationships between IFNs and DCs, since these cells can be a major source and/or target of these cytokines under various conditions.

The first section will synthesize current knowledge on IFN production and protective anti-viral functions. The I_2_R_2_s and downstream signaling pathways responsible for IFN-I production during viral infection will be listed. The roles of different cell types for this function will be discussed. The two main mechanisms through which IFN-I promote anti-viral defense will be reviewed, succinctly for direct anti-viral effects and in greater details for immunoregulatory functions.

The second section will focus on the detrimental functions of IFN-I. Selected diseases will be discussed to illustrate how different, and sometimes opposite, processes underlie deleterious IFN-I responses depending on the physiopathological contexts. IFN-I induction of unbridled inflammatory responses causing lethal tissue damage will be discussed as a major pathological mechanism during bacterial encounters secondary to influenza infection or in some autoimmune diseases. Inappropriate functional polarization of immune responses by IFN-I will be discussed as one potential cause for enhanced susceptibility to bacterial or fungal infections. The complex and disputed role of IFN-I in chronic viral infections will be reviewed, with emphasis on the physiopathology of the infections by human immunodeficiency virus type 1 (HIV-1) and human hepatitis C virus (HCV), with an outlook for the development of novel immunotherapeutic strategies to combine with anti-viral drugs.

The third section will recapitulate how the balance between beneficial versus deleterious IFN-I responses is modulated by several key parameters including (i) the source and timing of IFN-I production, (ii) the cell types affected by IFN-I, and (iii) the signaling pathways activated by IFN-I.

In the last section, we will speculate how integration of all the knowledge discussed before combined with advanced biochemical manipulation of the activity of the cytokines should allow designing innovative immunotherapeutic treatments, based on induction or blockade of specific IFN-I responses in targeted cell types. This “activity-by-targeting” concept is based on the design of novel “immuno-IFNs” consisting in covalent association between a mutated IFN-I with decreased affinity for its receptor and an antibody with high avidity for a molecule specifically expressed on target cell types (18). This design ensures lack of activity of the immuno-IFNs on all cell types but those targeted, contrary to previous strategies using IFNs with close to maximal potency that were still able to mediate strong off-target effects despite their coupling to cell-type specific antibodies and/or their local delivery.

## General Concepts on IFN Production and Functions

### How is the production of IFN controlled?

Type I interferons expression is not detectable under steady state conditions *in vivo* using classical methods such as gene expression analysis by RT-PCR or protein titration by ELISA or bioassays. However, mice deficient for the expression of the alpha chain of the IFN-I receptor (IFNAR1) harbor alteration in the ontogeny or functions of various cell types (19–26). Hence, extremely small or localized but functionally relevant quantities of IFN-I must be produced under steady state conditions (27). Indeed, the existence of steady state responses to IFN-I in various organs *in vivo* was demonstrated by using reporter mice expressing the firefly luciferase under the control of the promoter of *Ifnb1* (28) or of *Mx2* (29), a canonical IFN-I-stimulated gene (ISG). Steady state IFN-I responses are promoted by gut commensals (30). Early and transiently after many viral infections, large amounts of IFNs can be detected, in blood and spleen in the case of systemic infections or locally in the case of confined infections. IFN induction during viral infections results from the detection of specific danger signals by specialized I_2_R_2_s. This includes the detection of pathogen-associated molecular patterns as well as the sensing of stress signals or damage-associated molecular patterns (31, 32). Based on the nature and intracellular location of the danger signals that induce the production of the cytokines, the cellular sources of IFNs during viral infection can be classified in two main groups. Infected cells often contribute to IFN production as a response to their sensing of endogenous viral replication, or consecutive to the metabolic stress induced during massive translation of viral structural proteins, or as a result of plasma membrane perturbations upon viral entry. Specific subsets of uninfected cells can also significantly contribute to IFN production upon engulfment of material containing viral-derived nucleotide sequences and sensing of these molecules in endosomes by specific I_2_R_2_s. All sensing pathways leading to IFN induction converge on the activation of interferon response factors 3 or 7 (IRF3/7), which are the master transcription factors inducing IFN genes. Most cell types constitutively express IRF3 but not IRF7 or only at low levels. IRF7 expression requires IFN-I stimulation. IFN-β can directly be induced by IRF3. All but one of the IFN-α subtypes require IRF7 for their induction. Hence, IFN-β secretion promotes its own production and that of IFN-α in an autocrine manner (33, 34). This positive feedback loop strongly amplifies IFN production during viral infections, promoting fast and widespread induction of cell-intrinsic anti-viral defenses in uninfected cells to prevent virus dissemination. Other feedback loops tightly regulate IFN-I production positively or negatively. This section reviews different mechanisms controlling IFN production and how they could play different roles in host/virus interactions.

#### IFN production in infected cells is initiated by sensing of endogenous viral replication

##### Plasma membrane modifications occur upon virus entry which can induce IFN-I production and ISGs through a STING-dependent signaling

**Figure 1:**
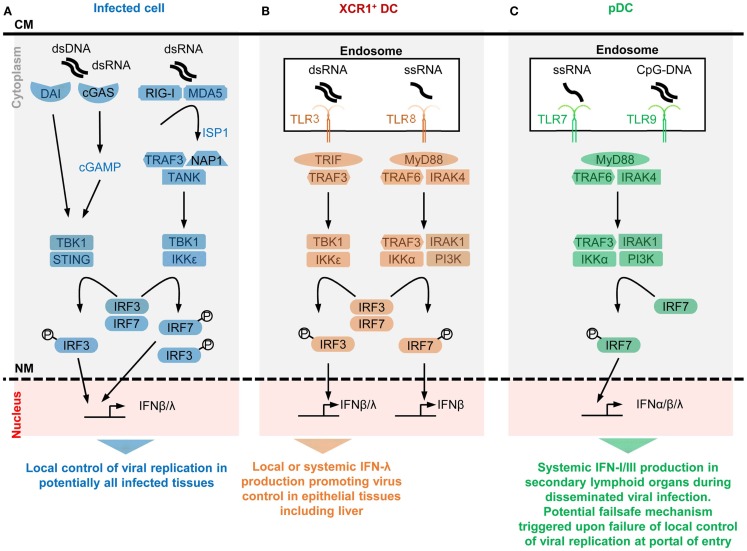
**A simplified model of the potential contributions of selective sensors and cell types to IFN production during viral infections**. Different innate immune recognition receptors are involved in sensing various types of viral nucleic acids in distinct categories of cells during viral infections, which may promote different types of anti-viral defenses. For each selected sensor shown, the types of viral nucleic acids recognized and the downstream signaling cascade induced are represented in a simplified, schematic manner. The potential specific role of each cell type in anti-viral defenses is also indicated at the bottom of each panel. **(A)** Potentially all types of infected cells can detect endogenous viral replication through cytosolic sensors triggering their local production of IFN-β/λ to control viral replication in an autocrine and paracrine fashion in infected tissues. **(B)** Uninfected XCR1^+^ DCs selectively produce high levels of IFN-λ and IFN-β upon engulfment of materials containing dsRNA and the consecutive triggering of TLR3 in endosomes. The receptor of IFN-λ is mostly expressed by epithelial cells. Hence, XCR1^+^ DCs might be involved in inducing local IFN responses in virally infected epithelial tissues. Since XCR1^+^ DCs are especially efficient at producing IFN-III upon HCV stimulation, they might contribute to local or systemic IFN production during infection with this virus, to promote IFN-λ-mediated protection of hepatocytes. Uninfected XCR1^+^ DCs and other uninfected cells may produce some IFN-β upon engulfment of materials containing ssRNA and the consecutive triggering of TLR8 in endosomes. The contribution of this pathway to anti-viral defense is not well understood yet, in part because mouse TLR8 is deficient for this function. **(C)** Uninfected pDCs selectively produce high levels of all subsets of IFNs upon engulfment of materials containing ssRNA or CpG DNA and the consecutive triggering of TLR7/9 in endosomes. However, pDCs seem to be activated for this function only in lymphoid tissues. Hence, pDC might contribute to systemic IFN production during blood-borne viral infections or as a failsafe mechanism activated upon abnormal widespread dissemination of a viral infection once it has escaped local confinement at its portal of entry. CM, cell membrane; NM, nuclear membrane.

Infected cells can sense abnormal changes in the physical or biochemical properties of their plasma membrane upon virus entry, which can trigger their production of IFN-I (35, 36). This event depends on signaling by the endoplasmic reticulum (ER) – resident transmembrane protein stimulator of interferon genes (STING). Upon virus entry, STING translocates to the cytosol where it is activated by phosphatidylinositol 3-kinase (PI3K) and calcium-dependent pathways to initiate a signaling cascade leading to IRF3-dependent induction of IFN-I and ISGs (Figure 1) (31, 37).

##### Viral nucleotide sequences are sensed by dedicated I_2_R_2_s in the cytosol of infected cells, which induces IFN-I production

Some I_2_R_2_s are located in the cytosol and bind viral nucleotide sequences to induce IFN-I production in infected cells. These I_2_R_2_s are classified as cytosolic RNA or DNA sensors. Their specificity for particular nucleotide sequences or tertiary structures, their signaling pathways and their physiological significance have been recently reviewed (31, 32). Cytosolic RNA sensors encompass DExD/H helicases among which the retinoic-acid-inducible gene (RIG)-I-like receptors (RLRs) have been the most studied, namely RIG-I and melanoma differentiation associated gene 5 (MDA5). RIG-I recognizes RNA with a 5′-PPP or 5′-PP (38) (uncapped) moiety, or double-stranded RNA (dsRNA), both structures being present in viral, but not in cytosolic eukaryotic, RNA molecules. MDA5 might specifically recognize long dsRNA fragments. Both RIG-I and MDA5 contain a DexD/H box-containing RNA helicase domain, and 2 caspase recruitment domains (CARD1/2), which bind to mitochondrial anti-viral signaling protein (MAVS). RNA/RLR/STING molecular complexes initiate a signaling cascade leading to IRF3/7-dependent induction of IFNs (Figure 1). Other DExD/H helicases can promote IFN-I production in DCs, although their physiological roles for *in vivo* immune defenses against viral infections remain to be established (32). Cytosolic DNA sensors able to induce IFN-I (mostly IFN-β) and IFN-III encompass molecules belonging to different protein families, including DExD/H helicases, the inflammasome component IFN-γ-inducible protein 16 (IFI16), the Z-DNA binding protein 1 (ZBP1), and the cyclic GMP-AMP (cGAMP) synthase (cGAS) (31, 32). Most of the cytosolic DNA sensors activate STING and lead to IRF3/7- and NFκB-dependent induction of IFN-β and IFN-III. Many cell types express ZBP1 and are able to produce IFN-I upon triggering of this molecule, including macrophages, DCs, and fibroblasts following an HSV-1 infection (39, 40). Upon DNA binding, cGAS catalyzes the production of cGAMP. cGAS is critical for the detection of lentiviruses including HIV-1/2 (41, 42) and can contribute to sensing of, and protection against, other RNA viruses, including *in vivo* in mice (43). cGAMP also acts as a secreted second messenger signal alerting uninfected cells to directly induce their expression of intrinsic immune anti-viral defenses. The cGAS/STING/IRF3 signaling cascade and the IRF1 transcription factor are “master” inducers of cell-intrinsic immunity able to control the replication of most DNA and some RNA viruses at least in part independently of IFNs (43).

##### Viral hijacking of the protein synthesis apparatus of the host cell triggers ER overload, a stress, which synergizes with cytosolic sensing to promote IFN-I production

Infected cells become a factory for production of viral particles. Hijacking of the translation apparatus of the host cell for massive production of viral structural proteins leads to an overload of the capacity of the ER for correct folding of newly synthesized proteins. ER overload induces a homeostatic response of the cell, the unfolded protein response (UPR). UPR aims at restoring normal ER functions by inhibiting translation. UPR activation in infected cells contributes to prevent viral replication, including through inhibition of the production of viral proteins, promotion of IFN-I production, and induction of cell suicide (44).

#### IFN-I production in uninfected cells is initiated by endosomal sensing of viral nucleotide sequences derived from engulfed virions or infected cells

Toll-like receptors (TLRs) are among the first and best characterized I_2_R_2_s. TLRs are transmembrane proteins with a leucine-rich repeat extracellular domain involved in ligand recognition and an intracellular toll/interleukin-1 receptor domain essential for signaling (45). Among the nine TLRs conserved between mouse and human, TLR3, TLR7, TLR8, and TLR9 are located in endosomes where they can detect the abnormal presence of nucleic acids such as occurs upon endocytosis of virions or of virally infected cell material. TLR3 recognizes dsRNA, TLR7/8 ssRNA, and TLR9 DNA sequences containing unmethylated cytidine-phosphate-guanosine (CpG) motifs. TLR fine specificity and signaling pathways have been reviewed recently (32) and are summarized in Figure 1. We will discuss the expression patterns and functions of endosomal TLRs with regards to IFN production in uninfected specialized immune cell types, pDCs and XCR1^+^ DCs.

##### Selective expression of TLR7, TLR9, and IRF7 in pDCs endows them with a unique ability to produce very high amounts of all subtypes of IFNs upon virus stimulation irrespective of their own infection

Plasmacytoid DCs uniquely produce very large amounts of IFNs in response to *in vitro* stimulation with many viruses, without being infected (46). IFN-I mRNAs represent up to 40% of all mRNAs in pDCs at the peak of their activation (47). *In vitro*, upon exposure to influenza virus, herpes virus type 1, cytomegaloviruses, or vesicular stomatitis virus, individual pDCs produce 100–1000 times more IFNs than total PBMCs, monocytes, MoDCs, cDCs, neutrophils, and fibroblasts (47–52). However, *in vitro*, high molarity infection of cDCs with certain viruses unable to inhibit IFN-I production in their target cells can also induce massive IFN-β secretion (53). pDCs produce high levels of all subtypes of IFNs, contrary to many other cell types including infected cells, which often preferentially produce IFN-β (46, 47). *In vivo*, pDC depletion during systemic viral infections leads to over 95% decrease of IFN-I production, while the total number of pDCs producing IFN-I (<100,000 in one mouse) is much lower than the total number of infected cells (54–59). This shows that *in vivo* also individual activated pDCs produce much more IFN-I/III than most other cell types, including virus-infected cells. The professional IFN-producing function of pDCs largely results from their high constitutive and selective expression of IRF7, TLR7, and TLR9 (Figure 1). These molecules are pre-associated in ready-to-signal complexes located in specialized endosomes specific to pDCs (60, 61). pDCs must also be equipped for efficient sensing and up-take of virions and virus-infected cells. The corresponding cell surface I_2_R_2_s remain to be identified.

##### Selective expression of TLR3 in XCR1^+^ DC endows them with a unique ability to produce very high amounts of IFN-β and IFN-III upon stimulation with dsRNA or HCV irrespective of their own infection

XCR1^+^ DCs are very potent for antigen-specific activation of CD8^+^ T cells, in particular through cross-presentation of exogenous antigens that they have captured from other cells and processed for association with class I major complex histocompatibility (MHC-I) molecules (62). In mice, XCR1^+^ DCs are crucial for the initiation of protective adaptive immune responses against tumors and a variety of viruses (63). Mouse and human XCR1^+^ DCs constitutively and selectively express high levels of TLR3 (Figure 1). They produce large amounts of IFN-III and IFN-β upon stimulation with a synthetic mimetic of dsRNA, Polyinosinic:polycytidylic acid (PolyI:C) (64, 65). Human XCR1^+^ DCs uniquely respond to stimulation with HCV by producing large amounts of IFN-III in a TLR3-dependent manner (66, 67), irrespective of their own infection.

#### Positive and negative feedback loops regulating IFN-I production

##### Positive feedback loops

In addition to IRF7 induction, other positive feedback mechanisms exist to amplify the production of IFNs rapidly after initiation of a viral infection as illustrated by the following selected examples. IFNs induce the expression of many cytosolic RNA/DNA sensors and of TLR7. This broadens the spectrum of host’s cell types able to detect endogenous viral replication for IFN induction. Induction of OASL by IFNs in human cells enforces RIG-I signaling, counteracting viral immune evasion genes interfering with this sensing pathway (68). The IFN-inducible ribonuclease L (RNaseL) generates viral and cellular RNA degradation products, which engage RLRs for amplification of IFN production (69, 70). The IFN-inducible Protein kinase R (PKR) stabilizes IFN-I mRNA (71).

##### Negative feedback loops

To prevent unbridled responses deleterious for the host, IFN activity must be tightly controlled including during viral infections. Several negative feedback loops exist to terminate IFN production, after anti-viral defenses have been activated. The ISG ubiquitin specific peptidase 18 (USP18) binds to IFNAR2, preventing it from recruiting signal transducer and activator of transcription 1 (STAT1). IFNs induce the expression of TAM receptor tyrosine kinases in DCs, monocytes, and macrophages. TAM receptors associate and signal in part through IFNAR1. They activate the suppressors of cytokine signaling-1/3 (SOCS-1/3). SOCS inhibit TLR and RLR signaling, thereby terminating IFN production (72). TAM receptor ligands, Gas6 and ProS, bind phosphatidylserine on dying cells and are produced by activated DCs and monocytes/macrophages. Thus, IFN induction of TAM inhibitory receptors on uninfected phagocytic immune cells could limit their propensity to produce the cytokines upon engulfment of dying virally infected cells. IFNs induce Tetherin on most cell types. pDCs express a receptor for Tetherin, leukocyte immunoglobulin-like receptor, subfamily A (with TM domain), member 4 (LILRA4). LILRA4 triggering on pDCs inhibits their production of IFN-I. Hence, through LILRA4 engagement by Tetherin, pDCs can monitor their efficacy at inducing an anti-viral gene expression program in neighboring cells through IFNs, and timely terminate their IFN production.

How positive and negative feedback loops integrate in time and space to promote optimal kinetics and intensity of IFN production in order to efficiently control viral infection without causing severe immunopathology is not completely understood. Positive feedback loops may occur very rapidly after initiation of viral infection to allow rapid secretion of high levels of the cytokines for fast and strong induction of anti-viral cell-intrinsic immunity. Negative feedback loops occur likely later to terminate the response and thus avoid chronicity of cytokine production and its ensuing deleterious effects.

#### What are the respective roles of infected versus uninfected cells in IFN production during viral infections?

##### IFN production by infected cells serves as first line of defense to block viral replication at his portal of entry in the body, while IFN production by uninfected pDCs might constitute a failsafe mechanism activated only when viral infection gets systemic

pDCs do not constitute the major source of IFN production upon local infections by several viruses in the lung or in the female reproductive tract. pDCs are dispensable for resistance against these infections (56, 73, 74). During pulmonary infection by Newcastle disease virus (NDV), IFN-I are produced locally in the lungs mainly by infected alveolar macrophages. Lung pDCs do not express the cytokines (73). Selective depletion of lung alveolar macrophages leads to systemic dissemination of NDV, and to a strong activation of pDCs for IFN-I production specifically in the spleen. Even in the case of systemic viral infections such as caused by intravenous injection of NDV or intraperitoneal injection of mouse cytomegalovirus (MCMV), pDC IFN production is confined to the spleen. It is not detected in other organs even those with high viral replication (59, 73). Hence, splenic pDCs are especially prone to high level IFN production upon systemic acute viral infections. pDCs located in non-lymphoid organs, in particular mucosal barrier tissues, appear to be inhibited for IFN production. Thus, IFN production by infected cells serves as first line of defense to block virus replication at its portal of entry in the body. IFN production by uninfected pDCs might constitute a failsafe mechanism mainly activated in the spleen when viral infection gets systemic (75). Under these conditions, to promote health over disease, the benefits for the host of producing high circulating levels of IFNs in order to induce widespread cell-intrinsic anti-viral defenses might prevail over the deleterious effects that this could cause on certain cell types or tissues. Indeed, pDCs are required for protection against HSV-2 and HSV-1 in mice only in systemic but not local infections (56). This observation is consistent with the crucial role of pDCs for protection of mice against systemic infection by mouse hepatitis virus (MHV), a fast replicating coronavirus (55). Conflicting results have been obtained on the role of pDCs during intranasal influenza infection (74, 76–78). A possible explanation is that pDC IFN production contributes to resistance to highly pathogenic influenza strains that might systemically spread from the lung early after infection, even if at low levels. Another intriguing observation is that IFNs are critical for host resistance to MCMV and that pDCs are the major source of IFNs in this infection model but are dispensable for virus control (54). Studies are ongoing to understand this apparent paradox. Patients bearing genetic mutations disrupting endosomal TLR signaling do not appear to suffer from life-threatening viral infections (79, 80), contrary to patients impaired in IFNAR signaling (4, 81). A notable exception is the specific susceptibility to severe herpes virus encephalitis in individuals’ deficient for TLR3 signaling (82, 83). However, contrary to extracellular TLR, endosomal TLR have evolved under strong purifying selection in human beings (84). Hence, while pDCs and endosomal TLR might have been required for protection of our species against viral infections in the past, this appears not to be the case anymore perhaps due to improved social, hygiene, and health care in modern society (75).

##### IFN production by uninfected pDCs or XCR1^+^ DCs might promote protection against viruses able to interfere with the signaling pathways inducing cytokine production in infected cells

Attesting to the importance of IFNs for anti-viral defense in vertebrates, many mammalian viruses encode immune evasion genes specifically inhibiting the production of IFNs in infected cells (39, 85). pDCs or XCR1^+^ DCs might be essential for IFN-dependent host protection against these viruses, because they are spared from the intracellular functions of viral immune evasion genes (75). To the best of our knowledge, MCMV does not encode for immune evasion genes inhibiting IFN production. However, MCMV manipulates IFN-I responses through specific inhibition of STAT1 functions in infected cells. Thus, pDCs might be dispensable for resistance against systemic MCMV infection due to sufficient levels of IFN production by infected cells locally in all infected tissues. Hepatocyte responses to IFN-III appear to play a critical role in human resistance to HCV. In infected hepatocytes, HCV induces the expression of cellular microRNAs binding to IFN-III mRNA and leading to its degradation. Uninfected XCR1^+^ DCs produce high levels of IFN-III *in vitro* upon HCV stimulation (66, 67). Hence, during acute HCV infection *in vivo*, XCR1^+^ DC may be a strong and early source of IFN-III not subjected to virus immune evasion strategies, therefore, contributing to protect naturally resistant individuals.

##### Altruistic suicide of subcapsular sinus macrophages in secondary lymphoid organs promotes strong IFN responses to control viral dissemination

In secondary lymphoid organs, a subset of macrophages is critical for the clearance of viruses from the lymph (86). These macrophages are located on viral entry routes, near to subcapsular sinuses where the afferent lymph drained from non-lymphoid tissues flows. Contrary to other subsets of macrophages, subcapsular sinus macrophages are highly susceptible to viral infection, because they constitutively express only low levels of effector molecules of cell-intrinsic anti-viral immunity and because their responses to IFNs are inhibited by their high constitutive expression of USP18. Subcapsular sinus macrophages rapidly become infected by viruses incoming from the lymph and produce large amounts of IFNs. This altruistic suicide prevents virus dissemination to other adjacent cell types and promotes the induction of innate and adaptive anti-viral immunity (87).

### How are IFNs promoting anti-viral immunity?

#### IFN direct anti-viral effector functions: induction of effector molecules of cell-intrinsic anti-viral immunity

**Figure 2:**
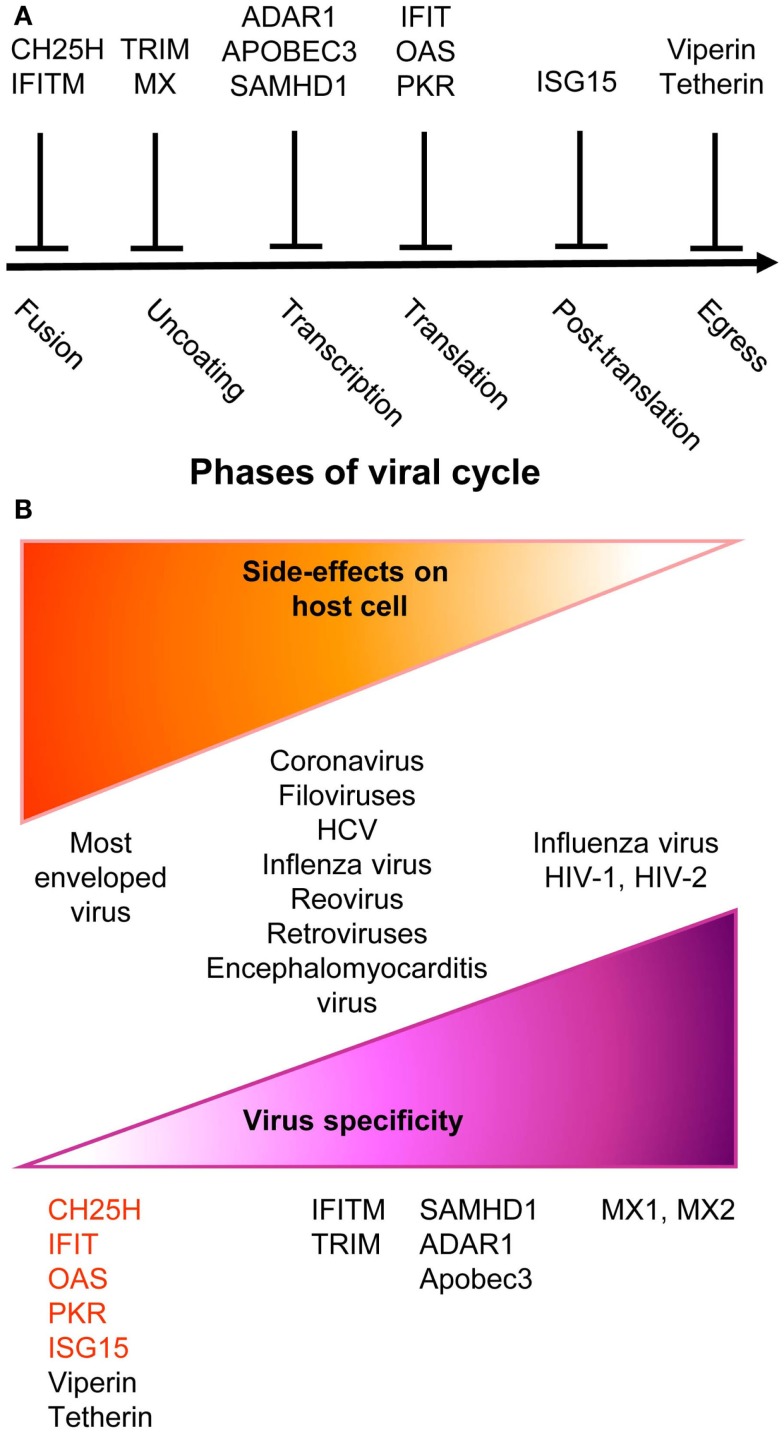
**A simplified and schematic view of the modes of action and specificities of ISG acting as direct anti-viral restriction factors**. **(A)** Different IFN-inducible restriction factors can block viral replication in infected cells in a cell-intrinsic manner at different stages of the viral life cycle. **(B)** Viral specificity (purple) might be inversely correlated to the breadth of side effects on host cells (orange).

Upon instruction by IFNs, cells express a wide variety of viral restriction factors, whose combined action blocks pathogen invasion by interfering with the different stages of viral life cycle (Figure 2A). This has been extensively reviewed recently (88) and will only be described succinctly here. Virus fusion with host cell membrane can be blocked by Cholesterol-25-hydrolase (CH25H) that inhibits sterol biosynthesis. Some viruses enter cells by escaping from endosomes/lysosomes, which can be blocked by interferon inducible transmembrane (IFITM) proteins. Virus uncoating can be blocked by tripartite motif (TRIM) proteins, such as TRIM5α, which bind to HIV-1 capsid thus promoting its degradation, and by Myxoma resistance GTPases, MX1, and MX2, which efficiently trap viral structural proteins at an early stage following virus entry into the cell. MX1 inhibits a number of viruses, including influenza virus through sequestration of its nucleocapsid. MX2 associates with host cyclophilin A and HIV-1 capsid protein. Virion assembly can be blocked at transcriptional, translational, and post-translational levels. The adenosine deaminase acting on RNA 1 (ADAR1) and the apolipoprotein B mRNA editing enzyme, catalytic polypeptide-like (APOBEC) deaminases induce viral RNA destabilization and hypermutation (89, 90). The sterile alpha motif and histidine-aspartic domain (HD) containing protein 1 (SAMHD1) blocks reverse transcription by hydrolyzing dNTPs (91). ADAR1, APOBEC, and SAMHD1 functions have been mainly studied in infections by HIV-1 and other retroviruses. The 2′,5′-oligoadenylate synthase (OAS) proteins, the IFN-induced proteins with tetratricopeptide repeats (IFIT), and PKR inhibit viral and host protein translation by using complementary mechanisms (88). The major post-translation modification induced by IFNs is the binding of the ubiquitin-like modifier ISG15 to several viral and host proteins, a process called ISGylation. Most of the known ISGylated proteins are targeted for degradation, with few exceptions that are on the contrary stabilized like IRF3 (88). Finally, the egress and budding of virions of many enveloped viruses can be inhibited by Tetherin or by Viperin (88).

Many anti-viral ISGs have been functionally characterized only recently, largely thanks to large-scale screening approaches. They display a variable degree of viral specificity (43, 92) that might inversely relate to the extent of their side effects on host cells (Figure 2B). Anti-viral effectors acting on a broad spectrum of viruses often target key metabolic pathways that are also crucial for host cell functions. This is the case for the control of cholesterol metabolism by CH25H (93) or of protein translation by PKR, OAS, or IFITs (88). Other anti-viral restriction factors such as MX2 may specifically target one molecule of a very restricted set of viruses with no apparent side effects on host cells. Some anti-viral ISGs target specific functions critical for only a restricted array of viruses and might similarly exert side effects only on a subset of host cell types. For example, SAMHD1 inhibits retrovirus replication through dNTP depletion, which might more specifically affect proliferating host cells. Hence, the infected host must balance the intensity, breadth, and location of ISG induction to circumvent viral replication while preventing life-threatening damages to vital cell types or tissues. One of the mechanisms contributing to this balance is translational control of the expression of ISGs, especially those with pro-apoptotic or anti-proliferative functions (94). While many anti-viral ISGs are transcriptionally activated in most IFN-stimulated cells, their translation can be specifically blocked in uninfected cells by cellular microRNA. This inhibition is relieved upon cell infection through negative regulation of the function of the RNA-induced silencing complex. Hence, IFN stimulation of uninfected cells prepares them for rapid and strong induction of cell-intrinsic anti-viral defenses upon viral infection while avoiding their unnecessary exposure to the toxic effects of certain ISGs.

Further knowledge on the functions and the dynamic regulation of ISGs is essential to develop novel therapeutic strategies against viral infections aiming at modulating IFN responses to promote their protective anti-viral cell-intrinsic functions over their deleterious toxic effects. A better understanding of the immunoregulatory effects of IFNs will also help.

#### IFN orchestration of anti-viral responses of both innate and adaptive immune cells

**Figure 3:**
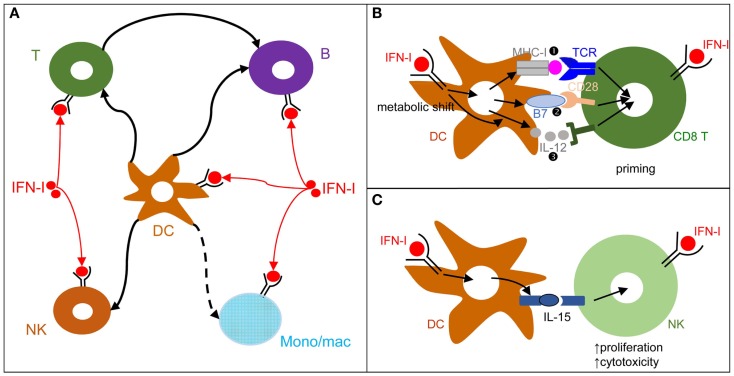
**DCs play a central role in IFN-I orchestration of innate and adaptive immune responses**. **(A)** IFN-I exert cell-intrinsic as well as indirect effects on a variety of immune cell populations. DC responses to IFN-I play a major role in promoting protective activation and functional polarization of other innate and adaptive immune cells, not only during viral infections but also in other physiopathological situations including cancer. **(B)** DC cell-intrinsic responses to IFN-I endow them to deliver appropriate signals for T cell priming and functional polarization. IFN-I can modulate all three types of signals delivered by DC to T cells: MHC-I/antigenic peptide complexes (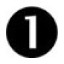), co-stimulation (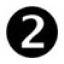), and cytokines (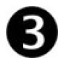). This depends both on IFN-I-dependent transcriptional induction in DC of some of the corresponding genes and on IFN-I-dependent metabolic reprogramming of DC. **(C)** DC cell-intrinsic responses to IFN-I endow them to deliver appropriate signals, in particular IL-15 trans-presentation, for NK cell activation. See main text for further details.

Type I interferon can modulate the functions of a broad spectrum of immune cells (Figure 3A). We will review this knowledge, focusing on the functions of DCs, NK cells, T cells, and B cells, since they are involved in the control of most viral infections. We will discuss the hypothesis that DCs play a central role in IFN-I orchestration of innate and adaptive immunity for the induction of optimal anti-viral defenses (Figure 3).

During viral infections and cancer immunosurveillance, IFN-I constitute one of the most important input signal acting on DCs to promote their delivery of appropriate output signals to T cells, B cells, and NK cells for protective immunity (Figure 3A). DCs deliver three types of signals to activate and functionally polarize T cells. Signal 1 is the triggering of the T cell receptor by viral peptide-MHC complexes. Signal 2 is the triggering of activating T cell co-stimulation receptors such as CD28 or CD27 by the CD80/86 and CD70 co-stimulation molecules expressed on DCs. Signal 3 corresponds to cytokines, which can promote T cell proliferation and acquisition of specific effector functions. Under steady state conditions, most DCs are in an immature state characterized by low level expression of MHC-II (signal 1) and co-stimulation molecules (signal 2) and by the lack of production of T cell-activating cytokines (signal 3). Upon activation, including early after viral infections *in vivo*, DCs up-regulate their expression of signal 1 and activating signal 2 and secrete T cell-activating cytokines. This process is called DC maturation. Gene expression profiling of DCs stimulated by microbial stimuli identified a core set of genes up-regulated in mature DCs irrespective of the stimulus they receive, irrespective of the subset they belong to, and conserved across evolution (95). Most of these genes are induced during DC maturation in part through cell-intrinsic IFN-I signaling (95). Consistently, cell-intrinsic IFNAR signaling in DCs is required in many circumstances for the induction of protective immunity, including efficient CD8 T cell responses during viral infection or tumor development (96–98), Th1 responses upon PolyI:C injection independently of IL-12 or IFN-γ effects (99, 100), as well as follicular helper T cell and humoral responses (101, 102). Mechanistically, IFN-I promote DC immunogenicity for efficient T cell activation through a variety of effects (Figure 3B). It drives DC up-regulation of signal 2 *in vivo* during viral infections (103) and boosts their capacity to cross-present antigens for increased delivery of signal 1 to CD8 T cells (96–98). It shapes their delivery of activating signal 3, in particular inducing IL-15 and promoting or inhibiting IL-12 depending on experimental conditions (58, 104). Finally, it is necessary to induce their metabolic shift from mitochondrial oxidative phosphorylation to aerobic glycolysis, which fuels the increased needs in energy and the expansion of the intracellular organelles required for the production and proper intracellular routing of the signal 1 and 2 proteins (100, 105). Selective inactivation of IFNAR on cDCs compromises mouse resistance to MCMV and MHV infections (103, 106). In contrast, IFNAR expression is not required on NK cells for protection against MCMV and on pDCs, T cells, and B cells for early control of MHV replication (103, 106). Although cell-intrinsic IFN-I signaling in NK cells can promote their activation (107) (Figure 3A), IFN-I-induced IL-15 trans-presentation by DCs plays a more prominent role for this function in many conditions including *in vivo* during MCMV infection (103, 108) (Figure 3C).

Cell-intrinsic IFN-I signaling in CD4 T cells (109), CD8 T cells (110, 111), and B cells (112) can also contribute to their efficient activation and functional polarization (Figure 3). This depends on experimental settings. CD8 T cell-intrinsic IFN-I responses are crucial for mounting efficient cytotoxic CD8 T cell responses against LCMV but are less critical against Vaccinia virus and vesicular stomatitis virus (110, 113, 114). Mechanistically, cell-intrinsic IFN-I signaling in CD8 T cells can promote their survival during their antigen-induced proliferation (110). Cell-intrinsic signaling in DCs and CD8 T cells may act in a synergistic manner. Indeed, conditional inactivation of IFN-I responsiveness was required to occur simultaneously in both of these two cell types to dramatically affect CD8 T cell expansion upon vaccination with a modified Ankara vaccinia virus (115).

In summary, IFN-I generally play a crucial, beneficial, role in immune defenses against viral infections, both through the induction of cell-intrinsic anti-viral defenses and through the orchestration of innate and adaptive immunity. However, if these responses are not properly regulated, they can contribute to diseases as we will now discuss.

## Different, and Sometimes Opposite, Processes Underlie Deleterious IFN-I Responses Depending on the Physiopathological Contexts

### Deleterious effects resulting from the induction of unbridled inflammatory responses causing severe tissue damage, as exemplified in autoimmune diseases

**Figure 4:**
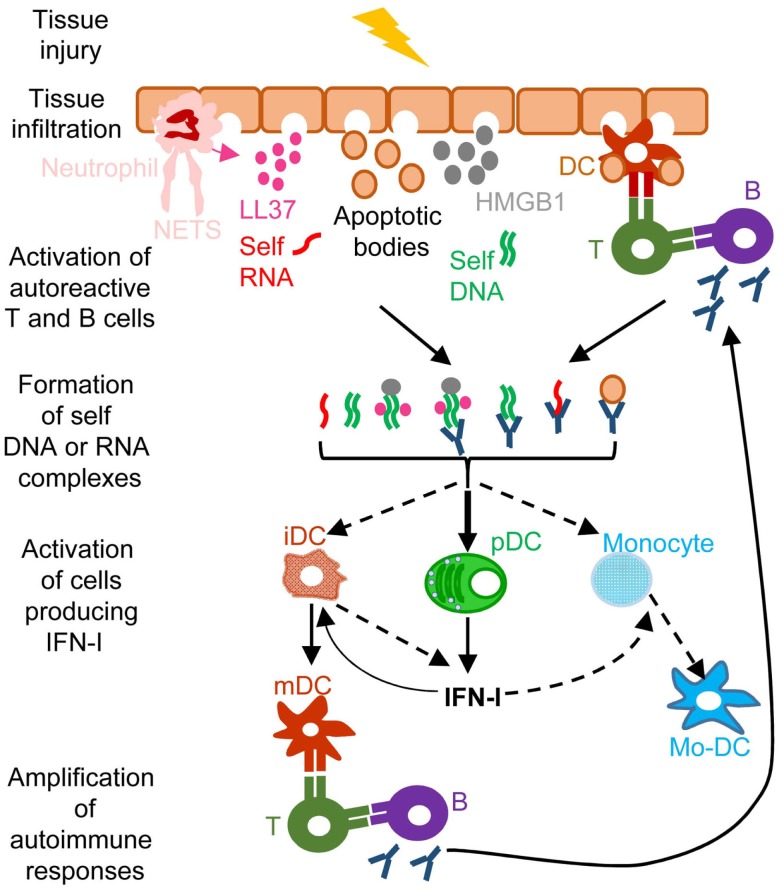
**A simplified model of the deleterious role of IFN-I in several autoimmune diseases**. When exposed to different kinds of injuries (microbial infection, commensal microbiota dysbiosis, chemical or physical insults), healthy tissues can undergo cell damage and death. These events induce the release of apoptotic bodies encompassing self RNA or DNA. Neutrophil recruitment and activation in inflamed tissues can also constitute a potent source of self nucleic acids, through the release of neutrophil extracellular traps (NET). Self RNA or DNA can associate with cationic peptides (e.g., LL37) as shown in psoriatic patients or with inflammatory molecules (e.g., high mobility group box 1, HMGB1) to generate nanoparticles that are extremely efficient for IFN-I production by pDC and eventually other cell types. pDC can also be efficiently activated for IFN-I production by immune complexes (ICs) generated by the association between self nucleic acids and auto-antibodies as frequently found in the serum of systemic lupus erythematosus patients. IFN-I promote the differentiation and/or the maturation of antigen-presenting cells, in particular different subsets of DC. Activated DC can then present self-antigens for activation of auto-reactive T CD4^+^ cells, including follicular helper lymphocytes, which in turn activate auto-reactive B cells for auto-antibody secretion, leading to a vicious circle of reciprocal activation between innate and auto-reactive adaptive immune cells. iDC, immature DC; mDC, mature DC; Mo-DC, monocyte-derived DC. See main text for further details.

A frequent side effect of IFN-I treatment against cancer or chronic viral infections is the induction of autoimmune reactions. Consistently, ISG expression is a hallmark of many spontaneous systemic or tissue-specific autoimmune diseases, including systemic lupus erythematosous (SLE), Sjogren’s syndrome, psoriasis, and other skin disorders (11). The dysregulation of IFN-I responses observed in patients with these autoimmune diseases likely results from both genetic and environmental factors. Genome-wide association studies show that polymorphisms in genes involved in IFN-I responses strongly correlate with increased susceptibility to many autoimmune diseases (11). Diverse environmental factors can also contribute to the onset of autoimmune diseases. Microbial infections often precede first clinical manifestations of autoimmune diseases. Whether infections (116) and/or alterations in the commensal microbiota of the affected barrier tissues (117, 118) are the cause or rather the consequence of autoimmunity is still matter of debate. Infection- or dysbiosis-induced tissue damages and unbridled IFN-I responses can contribute to initiate autoimmune reactions. Gender is another prominent factor affecting susceptibility to autoimmune diseases. Women are more prone to autoimmunity, which may result from endocrine regulation of IFN-I responses. pDC IFN-I production is enhanced in human and mouse females, due at least in part to cell-intrinsic enhancement of TLR7/9 responses by the female hormone estradiol (119). In autoimmune diseases, different mechanisms could operate to initiate the dysregulation of immune responses leading to a vicious circle of reciprocal activation between innate IFN-I responses and adaptive self-reactive lymphocyte responses (Figure 4). Adaptive immune cells are educated to spare “self.” This occurs through negative selection of potentially autoimmune B and T cells during their development in the bone marrow or thymus, respectively, a process called central tolerance. Self-reactive B or T cells that have escaped this pruning can be either deleted or functionally inactivated once they have egressed in secondary lymphoid organs or non-lymphoid tissues, a process called peripheral tolerance. In some individuals, polymorphisms in genes involved in the promotion of central or peripheral tolerance lead to a higher number, diversity, and/or responsiveness of self-reactive lymphocytes in the periphery, in particular of B cells secreting anti-DNA or anti-RNP antibodies (120, 121). Mammalian DNA or RNA are poor inducers of pDC IFN-I induction under normal conditions. However, pre-existing anti-DNA or anti-RNP autoantibodies can break this innate tolerance of pDC. Indeed, antibodies binding to self nucleic acids can protect them from degradation and compact them into nanoparticles that are very effective for the induction of IFN-I in pDC (Figure 4). DNA-containing immune complexes (ICs) are frequently found in the serum of SLE patients (SLE-ICs) and can activate pDC IFN-I production (122). In turn, pDC IFN-I activate cDCs, monocytes (123), and B cells, leading to a vicious circle of reciprocal activation between DCs and self-reactive lymphocytes and to the exacerbation of autoimmune responses (Figure 4). Certain infections or dysbiosis of the commensal microbiota of the affected barrier tissues could promote chronic production of host amphiphatic peptides able to combine with eukaryotic DNA or RNA, likely released from dying cells, thus forming pDC-activating nanoparticles. Indeed, in psoriatic skin, both a high expression of LL37 and a massive infiltration of pDCs is observed (124) (Figure 4). Hence, to treat many autoimmune diseases, novel therapeutic strategies could be designed to target dysregulated pDC IFN-I production or B cell activation by IFN-I.

### Deleterious responses resulting from inappropriate functional polarization of immune responses, as exemplified in failure to control secondary bacterial or fungal infections

**Figure 5:**
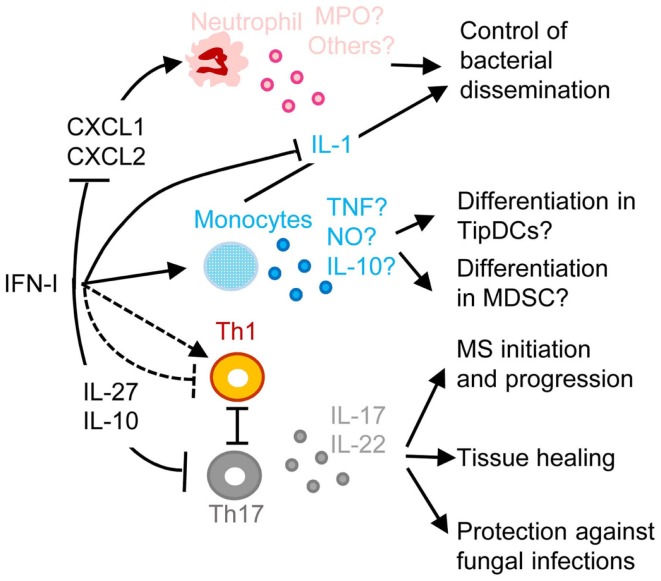
**A simplified model of the deleterious role of IFN-I in secondary pulmonary bacterial infections or in fungal infections and of their protective role in multiple sclerosis (MS)**. IFN-I-dependent signals can block CXCL1 and CXCL2 production, thus inhibiting the recruitment and activation of neutrophils in inflamed tissues, hampering their protective functions against secondary pulmonary bacterial infections or fungal infections. IFN-I-dependent signals can enhance CCL2 production, promoting the recruitment in inflamed tissues of CCR2^+^ monocytes that can potentially differentiate into TipDC or into MDSC. This can contribute to disease either through enforcing T cell activation leading to immunopathology or on the contrary through suppressing anti-microbial immune defenses. IFN-I can either inhibit or promote Th1 responses, which in the latter case occurs at the expense of Th17 responses thus compromising the production of IL-17 and IL-22, which are respectively required for control of microbial replication and for tissue healing. IFN-I-induced IL-10 and IL-27 can directly inhibit Th17. In MS, inhibition of Th17 functions may contribute to the protective effects of IFN-β therapy. MPO, myeloperoxidase; TNF, tumor necrosis factor alpha; NO, nitric oxide. See main text for other abbreviations and further details.

One of the most common complications of primary infections by many respiratory viruses, in particular influenza virus, is a life-threatening pneumonia due to secondary pulmonary infections by bacteria, such as *Streptococcus pneumoniae, Staphylococcus aureus*, or *Haemophilus influenza* (125, 126). These pathologies affect especially infants, elderly, and immunocompromised patients. Retrospective studies indicate that secondary bacterial pneumonia was highly recurrent in lung tissues isolated from patients who died during last century influenza pandemics, independently of antibiotic availability (127, 128). Influenza virus induces high IFN-I responses in human beings and mice. In both hosts, secondary bacterial infections are lethal only when they occur in a limited time window following primary viral infection (3–7 days), around the peak of IFN-I responses, before complete virus clearance. Mouse models of viral/bacterial coinfections are being used to dissect disease mechanisms (129). IFNAR1-deficient mice appear more resistant to secondary pulmonary bacterial infections, showing that IFN-I responsiveness contributes to disease (130). Similarly, after lymphochoriomeningitis virus (LCMV) infection, wild-type but not IFNAR1-deficient mice are more susceptible to LPS-induced septic shock (131). Several mechanisms may contribute to the detrimental role of IFN-I in secondary bacterial infections (Figure 5). Early during viral infection, IFN-I decrease the host ability to control bacterial replication, by dominantly polarizing immune responses toward anti-viral functions, simultaneously inhibiting the development of the types of immune responses required for protection against most bacterial infections. IFN-I can inhibit the production of chemokines required for the recruitment to the respiratory tract of antibacterial effector innate immune cells, in particular neutrophils or monocytes/macrophages (132, 133) (Figure 5). Depending on the experimental models used, IFN-I can on the contrary induce a CCR2-dependant recruitment of classical monocytes (134). In infected tissue, IFN-I might skew the functional polarization of resident or infiltrating monocytic cells toward immunosuppression, because it does limit their antibacterial functions by inhibiting their IL-1 production (135–137) while it might promote their production of IL-10 and nitric oxygen intermediates. The exact nature of infiltrating monocytic cells is not clear and could correspond to activated classical monocytes, MoDCs, monocyte-derived macrophages, or myeloid-derived suppressor cells (MDSCs). The boundaries between these putatively different cell types are currently ill-defined (138). These cells could fuel local replication of monocyte/macrophage-tropic bacteria (134), be immunosuppressive (139) or contribute to local immunopathology (140). The role of IFN-I on monocytes/macrophages is complex and will require further investigations to determine when it is protective versus deleterious and what the underlying mechanisms are. Depending on the context, IFN-I can either promote or inhibit the induction of Th1 cytokines such as IL-12 and IFN-γ, and myeloid cell responses to IFN-γ (10, 141-143). IFN-I can also polarize CD4 T cell responses toward Th1 at the expense of Th17, while the Th17-type cytokines IL-17A and IL-22 are required for host defense against pulmonary bacteria by inducing the production of anti-microbial peptides and of tissue repair molecules (Figure 5) (141–143). IFN-I may not only affect host resistance to bacterial infection, but also host tolerance, i.e., the ability of the host to tolerate a given burden of pathogen without undergoing excessive tissue damages (143, 144). Hence, to counter IFN-I deleterious effects during secondary bacterial infections, it will be important to better delineate the respective contribution of lung tissue tolerance modulation and of immune-mediated resistance weakening.

Another well documented example of deleterious effects of IFN-I due to their inappropriate functional polarization of immune responses is the enhanced susceptibility to fungal infections of patients with genetically determined hyperactive IFN-I responses, as exemplified in the hereditary disease Chronic Mucocutaneous Candidiadis (CMC) (Figure 5) (145). Patients with CMC have a significant deficit in Th17 CD4 T cells, at least in part as a consequence of altered responsiveness to IL-6 or IL-21. Several STAT1 mutations were identified in patients with autosomal dominant CMC. Gain-of-function STAT1 mutations were found to hard wire CD4 T cell responses to cytokines toward STAT1 signaling, compromising their STAT3-dependent ability to produce IL-17 upon IL-6 or IL-21 stimulation. This was associated to induction of a global IFN-I transcriptomic signature in blood (145). Deleterious IFN-I effects on immunity to *Candida* might not only occur in CMC patients but also in other types of individuals upon secondary fungal infections occurring shortly after a primary viral infection, likewise to the situation discussed above for secondary bacterial infections. Indeed, PolyIC induced IFN-I abrogate innate immunity to systemic candidiasis in mice (146), and IFNAR-deficient mice can be more resistant to *Candida* infection under certain experimental settings (147). However, the role of IFN-I in the modulation of the ability of immunocompetent hosts to control fungal infection is disputed (148, 149).

The inhibition of Th17 responses by IFN-I could be protective in at least one important human pathology, MS (Figure 5). MS represents a striking exception to the previously discussed detrimental role of IFN-I in autoimmune diseases. Indeed, a large proportion of MS patients have low serum IFN-I activity and low ISG levels. These MS patients present a significant reduction of MS relapse upon IFN-β administration (150). The underlying mechanisms are not yet completely unraveled. However, in the experimental autoimmune encephalitis mouse model of MS, Th17 responses bear a major contribution to nervous system damages and are inhibited by the IL-10 and IL-27 induced upon IFN-I administration (151).

In summary, IFN-I responses can be deleterious in autoimmunity by promoting a vicious circle of reciprocal activation between innate immune cells and auto-reactive CD4 T or B lymphocytes. IFN-I responses can also be deleterious upon secondary bacterial or fungal infections in the lung or the kidneys occurring shortly after a primary viral infection, by compromising the recruitment of anti-microbial innate effector cells and/or by preventing the proper functional polarization of immune responses. We will now discuss how IFN-I responses can also compromise host immune defenses against certain viruses and promote chronic infections.

### Deleterious responses resulting from the induction of immunosuppression, as exemplified in chronic LCMV infection

Different LCMV strains such as Armstrong and clone-13 (Cl13), respectively, lead to acute versus chronic infections in mice. A hallmark of chronic LCMV infection is the loss of the proliferative potential and effector functions of anti-viral CD8 T cells, a process called exhaustion. Exhausted CD8 T cells are characterized by a high expression of the inhibitory receptors PD-1, CTLA4, and LAG-3 (152). *In vivo* blockade of these inhibitory receptors can reverse T cell exhaustion and allow resolution of the chronic infection (152). IFN-I and ISGs are induced early after infection with all strains of LCMV, albeit to lower levels with those leading to chronic infection. This early IFN-I production is critical to limit viral replication (3). In models of acute infection, IFN-I responses rapidly return to normal, undetectable, levels, before viral replication is completely controlled. In contrast, ISG induction is maintained in chronic infection, including the expression of PD-1 ligands on APCs and of the immunosuppressive IL-10 cytokine, consistent with a prolonged expression of IFN-I albeit at low levels (13, 14). *In vivo* neutralization of IFN-I by antibody administration promoted resolution of chronic LCMV Cl13 infection, allowing the restoration of functional anti-viral CD8 T cell responses at least in part through CD4 T cell- and IFN-γ-dependent mechanisms (13, 14). During persistent LCMV Cl13 infection, chronic low level IFN-I production polarizes CD4 T cell responses toward T follicular helper (Tfh) rather than Th1 functions. Thus, chronic IFN-I responses promote enhanced anti-viral B cell responses but facilitate CD8 T cell exhaustion due to deficient CD4 T cell help, therefore contributing to host failure to prevent chronic infection (153). Strikingly, establishment of chronic infection by LCMV Cl13 could also be prevented by early administration of two shots of a high dose of exogenous IFN-I, at days 2 and 5 post-LCMV inoculation. This treatment allowed viral clearance by rescuing anti-viral CD8 T cell from exhaustion (154). Altogether, these studies show that the timing and duration of IFN-I production during viral infections is critical in determining how this response will impact the balance between the virus and the host. An early and robust but transient production of IFN-I promotes strong induction of cell-intrinsic viral restriction mechanisms as well as adequate polarization of adaptive anti-viral immune responses, which combined effects lead to viral clearance. In contrast, if the production of IFN-I is too low and/or too late, both viral replication and low IFN-I responses become chronic, their combined action leading to induction of immunosuppressive effects and to inadequate functional polarization of CD4 T cells. This results in CD8 T cell exhaustion and maintenance of chronic infection. Chronic viral replication and CD8 T cell exhaustion is also a hallmark of HIV-1 infection. We will now discuss the complex and disputed role of IFN-I in this disease.

### The complex and disputed role of IFN-I in HIV-1 infection

**Figure 6:**
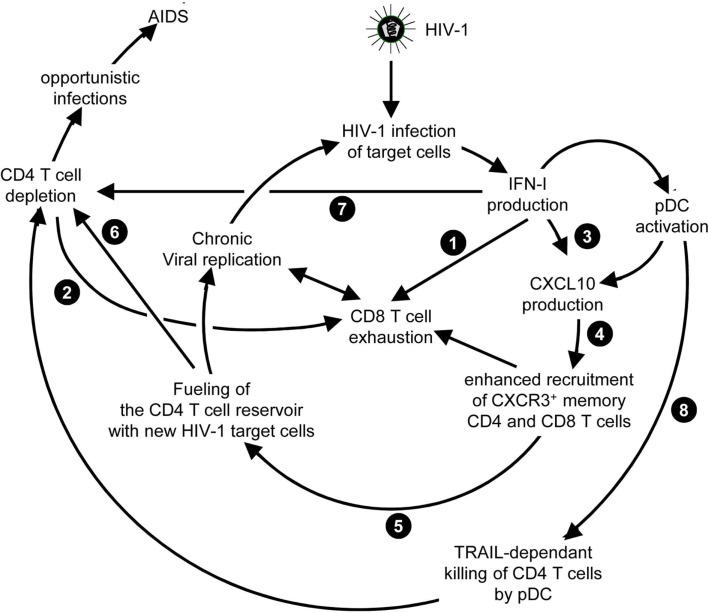
**Potential mechanisms through which chronic, low level IFN-I production might promote disease progression in HIV-1 infection**. High and sustained expression of ISG in blood and lymphoid organs is a hallmark of progressive infection with immunodeficiency viruses both in human beings and in non-human primates, irrespective of the levels of viral replication. Several mechanisms summarized here have been proposed to explain how chronic, low level IFN-I production might promote disease progression in HIV-1 infection. These mechanisms include direct (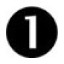) and indirect (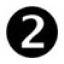) promotion of the exhaustion of anti-viral CD8 T cell responses, as well as direct (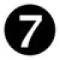) and indirect (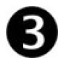-to-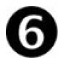, and 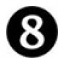) promotion of CD4 T cell depletion with a proposed central role of pDC in this deleterious process. Altogether, these mechanisms may sustain a vicious circle of reciprocal activation between chronic viral replication and deleterious immune responses, driving the progressive depletion of all CD4 T cells ultimately causing the enhanced susceptibility to opportunistic infections characteristic of the acquired immunodeficiency syndrome (AIDS). See main text for further details.

Both in HIV-1 infection and in its most relevant animal model, infection of non-human primates with simian immunodeficiency virus (SIV), disease progression after the acute phase of the infection is associated with high and chronic expression of ISGs while IFN-I production is inconsistently detected (155–157). In contrast, the individuals that do not progress toward disease despite persistent high viral loads show much lower immune activation, in particular low ISG expression, after the acute phase of the infection (158–161). Hence, chronic low levels of IFN-I are associated to disease progression independently of the level of viral replication. Therefore, an outstanding question still open for a better understanding of the physiopathology of HIV-1 infection is whether chronic IFN-I responses are merely a marker of progression, or whether they are implicated in driving disease development. In addition to mechanisms similar to those uncovered in the mouse model of chronic LCMV infection, during HIV-1 infection other effects of IFN-I could promote a vicious circle of reciprocal activation between chronic viral replication and sustained, deleterious immune responses (Figure 6). Very early after HIV-1 infection, in most individuals, IFN-I production might be too weak or too late to induce a combination of cell-intrinsic defense mechanisms and of immune responses efficient enough to prevent later establishment of chronic infection. On the contrary, as demonstrated in the case of the mouse model of LCMV infection, IFN-I responses could favor CD8 T cell exhaustion, either by direct cell-intrinsic effects on CD8 T cells (Figure 6, 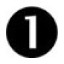) or by contributing to deprive them from CD4 T cell help (Figure 6, 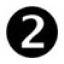). Several effects of IFN-I might compromise anti-HIV-1 Th1 responses or more generally contribute to the global depletion of CD4 T cells. These mechanisms include functional polarization of anti-HIV-1 CD4 T cells toward Tfh rather than Th1 responses, CXCL10 production leading to enhance recruitment of memory CD4 T cells to the sites of viral replication where they fuel chronic viral replication with new HIV-1 target cells (Figure 6, 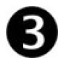 to 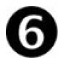), direct pro-apoptotic and anti-proliferative effects on CD4 T cells (Figure 6, 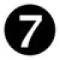), as well as TRAIL induction on pDCs licensing them for killing CD4 T cells irrespective of their infection (Figure 6, 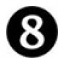) (162, 163). Altogether, these mechanisms entertain chronic viral replication and continuous depletion of CD4 T cells, leading to the dramatically enhanced susceptibility to opportunistic infections (Figure 6, 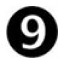) characteristic of the acquired immunodeficiency syndrome (AIDS) (Figure 6, 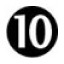). Other lines of evidences have been reported to support a deleterious role of pDC activation during HIV-1 infection. Women undergo faster HIV-1 disease progression than men with similar viral loads, which may result in part from the highest IFN-I production of women’s pDCs including in response to HIV-1 stimulation (164). pDC recruitment and activation in the vaginal mucosa of female macaques early after local SIV inoculation contribute to attract and activate CD4 T cells, which can then be infected and promote virus dissemination from its portal of entry (165). However, *in vivo* blockade of pDC IFN-α production by administration of TLR7/9-antagonistic oligonucleotides early after SIV infection of macaques did not decrease T lymphocyte activation, which suggests that additional sources of IFN-I likely contribute to the immune dysfunction observed in SIV/HIV-1 infections. Targeting dysregulated IFN-I responses during HIV-1 infection might represent an interesting adjuvant therapeutic strategy to highly active antiretroviral treatments. Administration of IFN-I in the non-pathogenic SIV infection model of sooty mangabeys was not sufficient to switch it into a pathogenic model. No CD4 T cell depletion ensued, no hyperactivation of immune responses were observed. Viral loads were even significantly decreased. However, this could be consistent with the positive impact of early and high dose IFN-I administration in chronic LCMV infection (154). Indeed, during the review process of this manuscript, it was reported that, early during primary SIV infection in the pathogenic rhesus macaque model, a high dose injection of IFN-I was protective while neutralization of endogenous IFN-I was deleterious. In contrast, in the same animal model, prolonged IFN-I administration accelerated disease development in the chronic stage of the infection (166). In mice with a humanized immune system, pDC depletion strongly decreased ISG induction and enhanced viral replication both in the acute and chronic phases of HIV-1 infection. However, pDC depletion during chronic infection decreased infection-induced T cell apoptosis and increased T cell numbers in lymphoid organs (167). These results further emphasize the dual role of IFN-I and pDCs in the physiopathology of HIV-1 infection. A strong and transient production of IFN-I early after infection benefits the host by lowering the set-point of viral replication during chronic infection. Sustained production of low levels of IFN-I during chronic infection contributes to immune dysregulation and CD4 T cell depletion. Further studies will be necessary to examine whether complementing standard-of-use antiretroviral drugs with pDC depletion, IFN-I neutralization, or selective inhibition of T cell responses to IFN-I could yield additional benefits to chemotherapy in non-human primates during chronic SIV infection.

IFN-I administration has been used for many years to treat another human chronic viral disease, HCV infection. Roughly, half of the patients do not show sustained virological responses (SVR). The treatment causes severe side effects in many individuals. New chemotherapeutic drugs very potent at blocking HCV replication *in vivo* have recently become available. Hence, whether IFN-I administration still constitutes a viable treatment against chronic HCV infection is being questioned (168, 169). We will now discuss this issue.

### IFN-I treatment of chronic HCV infection: balancing benefits for virus control with side effects strongly affecting patient’s quality of life

**Figure 7:**
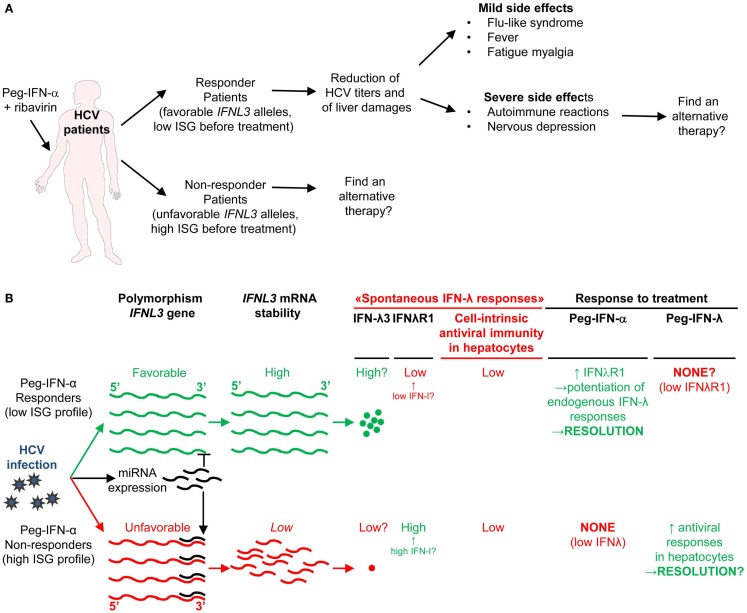
**A novel hypothetical model attempting to explain the respective roles of IFN-I and IFN-III in HCV infection**. **(A)** Classification of patients suffering from chronic HCV infection and treated with PEG-IFN-α in non-responders and responders, and identification of *IFNL3* (*IL28B*) gene polymorphisms as the best predictors for treatment response. The side-effects induced by PEG-IFN-α treatment are also listed since they can severely affect the patient’s quality of life and lead to treatment failure due lack of compliance or suicide. Hence, new approaches are needed to promote beneficial over deleterious effects of IFN-I administration in chronic HCV infection. **(B)** Proposal of a new hypothesis explaining the relationships between endogenous ISG levels in patients prior to treatment, *IFNL3* gene polymorphism, endogenous expression of IFN-I, IFN-λ, and IFNλR1, and responsiveness to IFN-I administration. Efficient control of HCV infection may depend on hepatocyte response to IFN-λ rather than IFN-α. Upon HCV infection, the virus induces the expression of host miRNA able to bind the 3′ UTR of *IFNL3* mRNA to promote their degradation. The favorable *IFNL3* allele associated with responsiveness to PEG-IFN-α treatment may allow endogenous expression of sufficient levels of IFNL3 for efficient induction of cell-intrinsic anti-viral defenses in hepatocytes. This process is, however, hampered by the limited expression of the receptor for this cytokine (IFNλR1) in these patients. PEG-IFN-α treatment might promote resolution of the infection by inducing IFNλR1 in these patients, potentiating their response to their endogenous production of IFNL3. In the patients that do not respond to PEG-IFN-α treatment, endogenous levels of IFNL3 are insufficient for efficient induction of cell-intrinsic anti-viral defenses in hepatocytes, due to the degradation of the corresponding mRNA in infected hepatocytes. In these patient’s hepatocytes, however, IFNλR1 is already expressed to high levels prior to treatment due to their high endogenous IFN-I responses. Administration of exogenous IFN-λ might cure these patients. See main text for further details.

Chronic HCV infection is the main cause of liver cirrhosis and hepatocellular carcinoma. There is currently no vaccine against HCV. The most common therapy for chronic HCV patients is the administration of recombinant pegylated IFN-α (Peg-IFN-α) combined with the anti-viral drug ribavirin. However, because of IFNAR pleiotropic expression, IFN-α administration induces severe side effects including flu-like syndrome, fever, fatigue, myalgia, and nervous depression (Figure 7A) (170). Moreover, only about half of treated patients harbor SVR (171). Prior-to-treatment high hepatic ISG expression is a negative predictor of SVR upon Peg-IFN-α therapy. High ISG expression in untreated patients likely results from chronic but low IFN-I production triggered by persistent HCV replication. Indeed, hepatocytes from non-responder patients were found to be infected at a greater frequency and to exhibit dampened antiviral and cell death responses (172). What the cellular sources of IFN-I production are and why they persist only in non-responder patients still remain to be established. In chronic HCV infection, cytotoxic effector lymphocytes may contribute to the development of hepatocarcinoma by causing low level but sustained hepatocyte death and renewal. In contrast, local production of IFN-γ in the liver by NK and T lymphocytes could promote resistance to disease through non-cytolytic control of viral replication. As discussed previously for LCMV and HIV-1, low chronic production of endogenous IFN-I in HCV patients could compromise both innate and adaptive anti-viral immune responses. Chronic exposure to IFN-I could dampen the ability of NK and CD8 T cells to produce IFN-γ (173, 174) and promote CD8 T cell exhaustion (175). It could also induce an antagonist form of CXCL10, a chemokine required for recruitment to the liver of anti-viral NK and CD8 T cell effectors (176). It may also polarize monocytes toward immunosuppressive functions (177). Therefore, better understanding IFN-I effects in HCV infection is critical to improve care of both responders and non-responder patients to Peg-IFN-α. For responder patients, the issue is to modify the treatment to favor beneficial antiviral and immunoactivating effects over side effects strongly affecting patient’s quality of life (Figure 7A). This might be achieved by specific delivery of IFN-I to targeted cell types as discussed later. For non-responder patients, the issue is to understand the mechanisms underlying treatment failure to determine whether alternative therapies could be designed (Figure 7A).

Genome-wide association studies identified various single nucleotide polymorphisms (SNP) in the gene encoding IL-28B/IFN-λ3, one of the IFN-III, as well in its 5′ and 3′ non-coding regions (178–181). One SNP, called rs12979860, is located 3 kb upstream of the *IFNL3* gene. Patients harboring the CC genotype have a favorable prognosis to IFN-I treatment. Patients with the TT genotype are at high risk of treatment failure (178, 179). In Europeans, the favorable CC genotype is the major, most common, *IFNL3* allele. The unfavorable TT SNP is the minor allele. The frequency of these alleles is reversed in Africans. The favorable allele allows escape of *IFNL3* mRNA from degradation by cellular microRNA induced upon HCV infection (181).

Until recently, *IFNL3* genotypes and hepatic ISG expression were considered as independent predictors of response to Peg-IFN-α treatment in HCV patients (171). Here, we propose a potential explanation, which integrates both factors in a relatively simple model (Figure 7B). Our main hypothesis is that efficient control of HCV infection depends on hepatocyte response to IFN-λ rather than IFN-α. This is supported by reports that IFN-λ induces a stronger and more sustained ISG expression in hepatocyte cell lines *in vitro* (182), and that PolyI:C-induced control of HCV replication in humanized liver in chimeric mice is correlated to the induction of IFN-λ but not IFN-I in human hepatocytes (183). Responder patients harboring the favorable *IFNL3* allele preventing the degradation of the corresponding RNA in infected cells might express significant levels of endogenous IFN-λ3, although this is disputed. However, they express only low levels of IFN-λR1, which limits IFNλ3 efficiency (Figure 7B) (184). How these patients benefit from Peg-IFN-α treatment could be that it induces IFN-λR1 expression on hepatocytes thus boosting endogenous IFN-λ3 effects (184). In contrast, high ISG-expressing non-responder patients harboring the unfavorable *IFNL3* allele might not express enough IFN-λ3 for virus control. However, they do express IFN-λR1 as a result of their endogenous production of IFN-I. Hence, Peg-IFN-α might be ineffective in these patients because they already express IFN-λR1 but fail to produce endogenous IFN-λ3 due to the degradation of its mRNA in infected hepatocytes (Figure 7B). These patients may be good candidates for Peg-IFN-λ therapy, currently undergoing clinical development. Since the expression of IFN-λR1 is mainly restricted to epithelial cells, melanocytes, and hepatocytes, some of the side effects related to IFN-I treatment might be strongly attenuated in Peg-IFN-λ therapy. However, as IFN-I are key to induce anti-viral immune responses, it will be critical to determine whether, beside viral clearance, Peg-IFN-λ therapy can also induce long-term immune protection against HCV.

## Cellular and Molecular Mechanisms Determining the Beneficial Versus Deleterious Outcome of IFN-I Effects

### Existence of different pathways downstream of IFNAR signaling

IFN-I transduce intracellular signals through a single receptor, IFNAR, but via a multitude of downstream signaling pathways. The Janus activated kinase (JAK)/STAT pathway was the first to be identified (185). IFNAR is composed of two distinct subunits, IFNAR1 and IFNAR2, which are constitutively associated with members of the JAK family, tyrosine kinase 2 (TYK2) and JAK1, respectively (186). The binding of IFN-I to their receptor leads to the phosphorylation of JAK1 and TYK2, which in turn induce the phosphorylation and activation of the STAT proteins (186).

**Figure 8:**
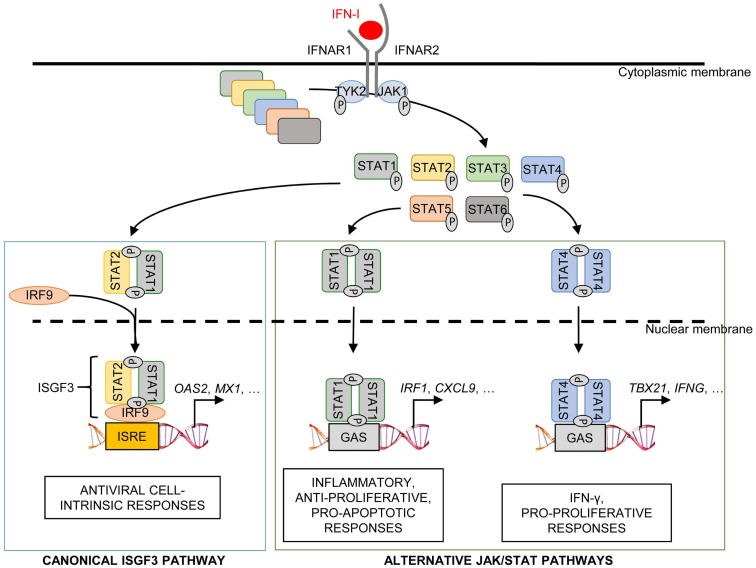
**Schematic representation of the ISGF3 and alternative JAK/STAT signaling pathways induced by IFN-I**. The receptor for IFN-I, IFNAR, is composed of two chains, IFNAR1 and IFNAR2, which are respectively associated with the JAK family kinases TYK2 and JAK1. IFN-I binding to IFNAR triggers the phosphorylation of TYK2 and JAK1, which in turn phosphorylate a variety of STAT proteins. Activated STATs are able to form complexes, as homo- or hetero-dimers. The heterodimer STAT1-STAT2 binds to a third partner, IFN-regulatory factor 9 (IRF9), in order to form the ISGF3 complex. This complex translocates into the nucleus and binds to specific regulatory sequences, IFN-stimulated response elements (ISRE), to activate the expression of many interferon-stimulated genes (ISGs). In particular, ISGF3 induces most, if not all, of the ISGs encoding effector molecules of cell-intrinsic anti-viral defenses such as OAS or MX1. Alternative JAK/STAT pathways include the formation of STAT1 or STAT4 homodimers, which may drive different functional responses to IFN-I. STAT1 homodimers bind to IFNγ-activated sequences (GAS) in the promoter of certain ISGs, which may promote inflammatory, anti-proliferative, and pro-apoptotic responses. STAT4 homodimers also bind to GAS but promote IFN-γ production and pro-proliferative responses.

Different STAT complexes can form upon triggering of IFNAR (Figure 8). A transcriptional complex that forms in most conditions of IFN-I stimulation and induces the expression of many molecules of cell-intrinsic anti-viral immunity is interferon-stimulated gene factor 3 (ISGF3), a heterotrimer composed of pSTAT1, pSTAT2, and IRF9 (187) (Figure 8). Following its translocation into the nucleus, ISGF3 binds to ISRE regulatory sequences in target genes. Many molecules playing a key role in the function of innate or adaptive immune leukocytes are also induced by ISGF3, including CD80, CD86, or IL-15 in DC, and Granzyme B in NK cells. ISGF3 is generally composed of STAT1 phosphorylated on Tyr701 and Ser727 and of STAT2 phosphorylated on Tyr689. However, alternative ISGF3 complexes have been described in various contexts which could participate to the diversity of IFN-I effects (188).

The pSTAT1 homodimer also plays a prominent role in cell-intrinsic IFN-I-dependent gene induction. It binds IFNγ-activated sequences (GAS) and controls the expression of many pro-inflammatory molecules (187). pSTAT1 homodimers can form upon stimulation with either IFN-I or IFN-γ. Many GAS-regulated genes can be induced by either cytokines.

Depending on cell types, JAK signaling downstream of IFNAR can lead to the activation of virtually all STAT proteins and to their combinatorial association into a variety of complexes with different affinities for specific GAS elements (189–191) (Figure 8). This diversity contributes to IFN-I induction of different transcriptional programs in distinct cell types (39). STAT complex formation depends in part on the relative abundance of STAT molecules in the cell (192). While STAT1, STAT2, STAT3, and STAT5 can be activated in most cell populations, STAT4 and STAT6 are mainly activated in lymphocytes (193). For example, quiescent NK cells express more STAT4 than STAT1, leading to constitutive association of IFNAR to STAT4 in these cells. Hence, quiescent NK cells mount pSTAT4 homodimer-dependent responses to IFN-I stimulation, including IFN-γ production and T-bet-driven proliferation (Figure 8) (194, 195). Changes in STAT levels can also occur upon the differentiation/activation of a given cell type and lead to a shift in its functional response to the cytokines (196). Upon activation, NK cells decrease their expression of STAT4 and increase that of STAT1, shifting their IFN-I response from STAT4-dependent in a quiescent state to STAT1-dependent in pre-activated cells. This translates into opposite IFN-I effects on IFN-γ production and proliferation for quiescent versus pre-activated NK cells (194). However, this outcome can be modulated by simultaneous exposure to other cytokines such as IL-15 or IL-12/18. A reverse STAT1-to-STAT4 shift occurs in DC during their maturation, shifting their functional responses from inhibition to activation of IL-12 production in response to combined stimulations with IFN-I and CD40L (197). This enables mature DC to efficiently activate CD8 T cells. Other yet unknown mechanisms control the formation of different STAT complexes in distinct cell types. The nature and dynamics of the signaling pathways triggered by IFN-α or -β were evaluated in bulk cultures of human blood leukocytes using flow cytometry (191) or high throughput mass cytometry (190). A diversity of phosphorylation patterns of STAT1/3/5 was observed upon IFN-I stimulation. IFN-α activation induced phosphorylation of STAT1, STAT3, and STAT5 in most cell types, peaking at 15 min (190). IFN-β-induced STAT1 phosphorylation was found to be poor in B cells as compared to monocytes and T cells (191). However, the underlying mechanism remains to be identified since B cells did not express lower amount of IFNAR2 or STAT1 or enhanced levels of the inhibitory SOCS1 molecule. The high STAT1 activation in monocytes led to their induction of IFN-I-dependent pro-apoptotic genes while this was not the case in B cells. These results strikingly differ from those obtained in the other study upon IFN-α stimulation, where STAT1 phosphorylation was on the contrary lower in CD14^+^ monocytes and was prolonged in B cells and NK cells (190). The differences between these two studies might have resulted from the use of different subsets and doses of IFN-I. In any case, both studies consistently reported that CD4 T cells showed the highest activation of STAT5. All CD4 T cells but not all CD8 T cells activated STAT5 and for a longer time (190). IFN-β activation of STAT3 was delayed in CD4 T cells and B cells as compared to CD8 T cells and monocytes (191). Different STAT complexes may lead to distinct transcriptional programs linked to different biological functions (Figure 8). More systematic studies are needed to understand this complexity. Besides changing STAT levels between cell types or activation states, the processes controlling differential formation of STAT complexes downstream of IFNAR triggering remain to be identified.

In addition to JAK/STAT signaling, other pathways can be activated downstream of IFNAR, including those involving the phosphatidylinositol 3-kinase (PI3K), mitogen-activated protein kinases (MAPK), and the CRK adaptor molecules (39, 198). This leads to the activation of other transcription factors such as IRF, NF-κB, or PU.1, which contribute to orchestrate cell responses to the cytokines by regulating both distinct and overlapping sets of genes as compared to STAT (199, 200).

In summary, IFNAR signals through a remarkable diversity of pathways, including but not limited to diverse combinations and kinetics of STAT phosphorylations. This explains at least in part the diversity of IFN-I effects, including their induction of opposite responses depending on the physiopathological contexts and/or the nature of the principal responding cell types (200, 201). IFN-III induce the same signaling pathways as IFN-I, although they engage a different heterodimeric receptor, composed of the IL-28RA and IL-10RB chains and preferentially expressed on epithelial cells including hepatocytes.

### The different IFN-I subtypes have different affinities for their receptor leading to their induction of distinct signaling

In mice and human beings, numerous IFN-I subtypes exist. Functional and population genetic analyses showed that these IFN-I subtypes significantly differ in their functions (202–207). Hence, one of most extraordinary feature of IFN-I biology is how IFN-I subtypes can elicit so many pleiotropic and diverse functions by interacting with the same receptor complex (208).

**Figure 9:**
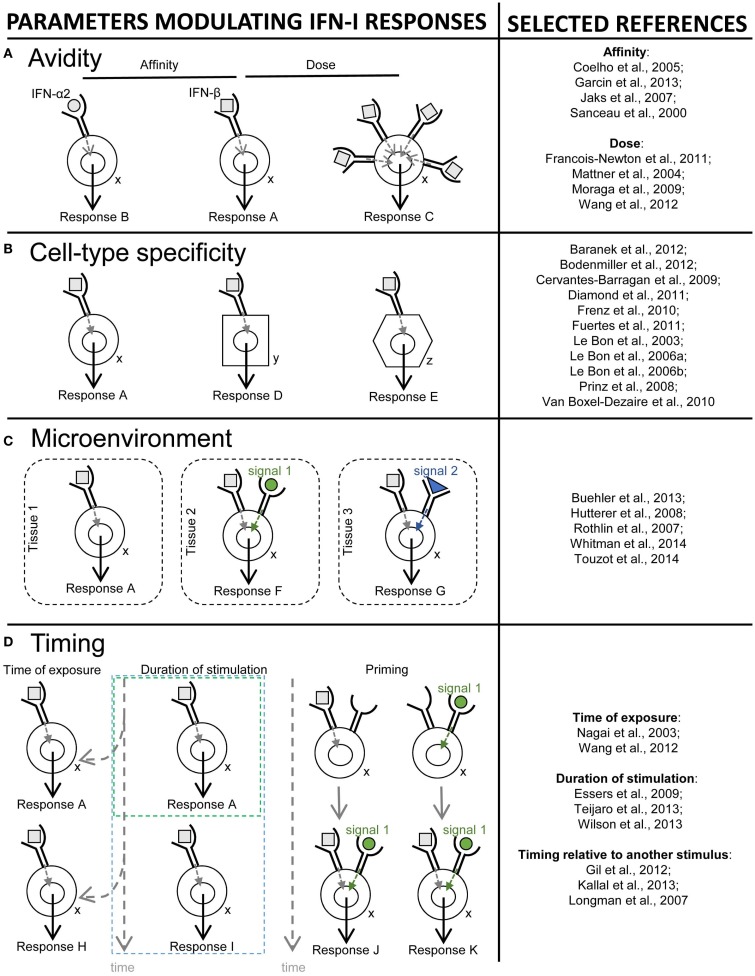
**Schematic illustration of different mechanisms controlling the diversity of IFN-I effects**. Different parameters that contribute to promote and control the diversity of IFN-I responses are depicted on the left side of the figure. References of papers illustrating each mechanism are given on the right side of the figure. **(A)** Avidity (a combination of affinity and dose). For example, the affinity of IFN-β for IFNAR1 is 100-times higher than that of IFN-α2, and IFNβ is much more potent in inhibiting cellular proliferation or monocyte differentiation into osteoclasts (response A), while both IFN-I subtypes are equipotent in establishing an anti-viral state (response B). The same subset of IFN-I can also exert different biological effects at low versus high doses. For example, low, but not high, doses of IFN-β protect BALB/c mice from progressive cutaneous and fatal visceral disease after *Leishmania major* infection. **(B)** Cell type specificity. Mouse DC but not NK cells are strong responders to IFN-I, and cell-intrinsic responses to IFN-I are critical in DC but not in some other cell types for immune defenses against viral infections or tumors. **(C)** Tissue microenvironment. The response of a given cell type to a given dose of a specific subset of IFN-I can also be modulated by the microenvironment of the cell. For example, in cancer, protective IFN-I effects on infiltrating DC or other immune cells might be dampened by inhibitors of IFNAR signaling locally produced by the tumor, such as ligands of the TAM receptor tyrosine kinases. **(D)** Timing. Differences in the time and duration of exposure to IFN-I can also determine distinct functional outcomes. For example, during viral infections, early and transient high levels of IFN-I promote protective DC and T cell responses, while delayed, chronic and low level IFN-I production compromises host immune defenses and promotes chronic viral infections. Within a given cell type, the outcome of IFN-I stimulation also depends on time of exposure to these cytokines relative to other modulatory signals (timing relative to other stimuli). For example, in naïve CD8 T cells, TCR signaling prior to IFN-I stimulation leads to increased expression of STAT4 and promotes IFN-γ production and proliferation, while IFN-I stimulation prior to TCR triggering leads to STAT1-dependent anti-proliferative and pro-apoptotic effects.

Both IFNAR1 and IFNAR2 are required for the initiation of IFN-I-dependent signals, as mice deficient in either one are highly susceptible to viral infections (3, 5). The assembling of the IFN-receptor ternary complex is a two-step process. First, a binary complex is formed by the binding of one side of the IFN molecule to IFNAR2. Then, a single binary complex interacts with IFNAR1 via the other side of the IFN molecule. The stability of the ternary complex will be determined in part by the association and dissociation kinetics between the cytokine and the two receptor chains, as well as by IFNAR expression levels since the cell surface concentrations of the receptor subunits are relatively low. Hence, both the affinity of IFN-I subsets for IFNAR and the amounts of IFN-I, IFNAR1, and IFNAR2 will regulate their biological effects (Figure 9A) (209, 210). Cell membrane density of IFNAR1 and IFNAR2 is also involved in differential IFN-β- versus IFN-α-induced functional activities, such as anti-proliferative function (211). A variety of cell-intrinsic parameters can also impact the lifetime of the IFN-receptor ternary complex, such as the rate of endocytosis/degradation/recycling of signaling complexes, and negative ISG regulators such as USP18 that decrease the affinity of IFN-IFNAR1 binding (203, 212).

Based on a definition of a prototypic cytokine-receptor binding module and by analogy with the EPO receptor system, IFN-I subtypes were originally postulated to form ternary complexes of differing architectures, resulting in distinct geometry and assembling of intracellular signaling components (213). Experimental evidence rejected this hypothesis. Rather, the differential activities of IFN-I subtypes are determined by the stability of the ligand/receptor ternary complex (207, 212). Differential affinities of the IFN-I subtypes for IFNAR1 and IFNAR2 extracellular domains generate subtype-specific signaling cascades and biological outcomes (Figure 9A) (210, 214). Crystal structure of ternary IFN-I/IFNAR1/IFNAR2 complex illuminated the biochemical complexity of IFN-I interaction with their cognate receptors (215). The main conformational features of IFN-I/IFNAR1/IFNAR2 ternary complexes are conserved among the different IFN-I, but are quite different from the other cytokine receptors (214, 215). In the formation of the binary IFN-I/IFNAR2 complex, IFN-I ligand discrimination resides on differential energetics during the interaction of anchor points with IFNAR2, shared by all IFN-I, as well as on key amino acid substitution among IFN-I subtypes (215). IFNAR1 then performs major conformational changes to interact with IFN-I associated in the binary complex, thus displaying an optimized functional plasticity (215). These differences in the chemistry of IFN-I subtype interaction with IFNAR2 and IFNAR1 thus explain the different affinities of IFN-α versus IFN-β within ternary complex and their differential activities (210).

### Cell-intrinsic responses of distinct cell types differentially contribute to IFN-I effects in various physiopathological conditions

The functions regulated by IFN-I strongly depend on the main responding cell types (Figure 9B). This has been studied *in vitro* by examining the functional consequences of the stimulation of different cell types with IFN-I, and *in vivo* by determining the contribution of cell-intrinsic IFN-I responses of different cell types to resistance or susceptibility to various diseases. An emerging concept is the central role of DC responses to IFN-I for induction of protective immunity against viral infections or tumors (Figure 3). The development of mutant mice allowing conditional genetic inactivation of *Ifnar1* in a cell-type specific manner using the Cre-lox system (216) has been instrumental in accelerating our understanding of how different cell types respond to IFN-I *in vivo* and what their respective contribution is to protective or deleterious IFN-I responses. This has been investigated most extensively in viral infections (106, 111, 112, 115, 217) but also in cancer (97, 98), bacterial infections (218), autoimmunity (216, 219), sepsis (220), or inflammatory diseases (221). Efforts are being pursued to better understand which cell types respond to IFN-I in a manner promoting protective versus deleterious effects in different physiopathological settings. That knowledge will considerably help to develop novel strategies to modulate IFN-I functions for promoting health over disease. The development of mutant mice allowing conditional genetic inactivation of *Stat1, Stat3*, and *Stat5* (222–226) will help better understanding how different signaling pathways in different cell types determine the outcome of IFN-I response *in vivo* in various conditions. This knowledge might lead to the development of strategies aiming at targeting a given cell type with a specific subset of IFN-I, or in the presence of antagonists of certain signaling pathways, to surgically tune IFN-I responses *in vivo* toward the most desirable outcome.

### The anatomical location and timing of cell exposure to IFN-I might also be major parameters controlling the effects of the cytokines

The formation of specific STAT complexes is a highly dynamic process. It depends not only on the cell type but also on its specific state at the time it sees IFN-I. Hence, major parameters controlling the effects of IFN-I in a given cell type also include its microenvironment (Figure 9C) and the timing of its exposure to the cytokines both in terms of duration of the stimulation and of previous activation history (Figure 9D).

The TAM receptor ligand Gas6 is expressed within tumor cells in various solid cancers (227, 228). Elevated Gas6 expression is of bad prognosis in different cancers (228, 229). In a mouse model of ovarian cancer, early during tumorigenesis tumor-infiltrating DCs were found to be immunogenic and promote antitumor immunity, but they were later altered in the course of tumor development to acquire immunosuppressive properties beneficial to the tumor (230). One may thus hypothesize that expression of TAM soluble ligands in certain tumors and of TAM receptors on tumor-infiltrating DCs might contribute to dampen DC response to IFN-I and therefore facilitate their polarization by the tumor microenvironment into immunosuppressive cells (Figure 9C).

Acute versus chronic exposure to IFN-I can lead to strikingly opposite effects on a given cell type (13, 14, 231). In addition to duration, the time when a cell is exposed to IFN-I can also dramatically impact its functional response, depending on its previous activation history (Figure 9D). *In vitro* stimulation of DCs with IFN-β can lead to opposite outcomes depending whether it occurs simultaneously to, or after, TNFα-induced maturation. IFN-β polarizes DCs toward Th1 induction in the former case, and toward IL-10-secreting T cells in the latter case. These opposite effects result at least in part from the differential expression of IL-12/18 by DCs (232). Similarly, IFN-I effect on the functional polarization of CD4 T cells is strongly modulated by the other cytokines present in the lymphocyte microenvironment at the same time (233). IFN-I can also mediate opposite effects on CD8 T cells depending whether it occurs before or after cognate engagement of the T cell receptor. Indeed, while CD8 T cells have the potential to respond to IFN-I by inducing both STAT1- and STAT4-dependent genes, this depends upon their activation history. Naïve CD8 T cells respond mostly to IFN-I through STAT1 signaling, leading to the inhibition of their proliferation and eventually to the induction of their apoptosis. However, cognate triggering of the T cell receptor causes a decrease in STAT1 and an increase in STAT4 expression in CD8 T cells. This leads to a shift of their IFN-I response from STAT1-to-STAT4 signaling, resulting in the promotion of their proliferation and IFN-γ production. During LCMV infection, this mechanism promotes STAT4-dependant expansion of anti-viral CD8 T cells, but STAT1-dependant inhibition of naïve CD8 T cell proliferation (234).

## Innovative Biochemical Engineering Approaches to Tune IFN-I Effects

**Figure 10:**
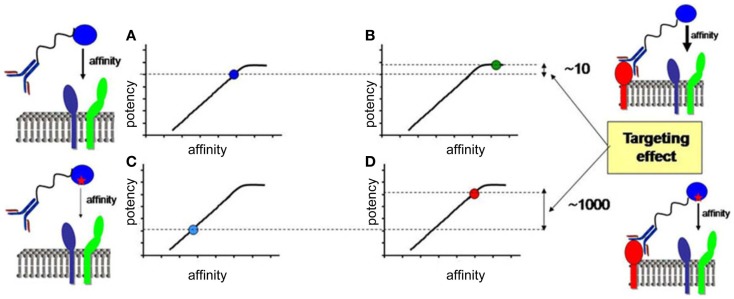
**Strategy for high efficiency cell type-specific targeting of cytokine activity**. **(A)** Most cytokines have evolved to exhibit optimized specific activities. **(B)** When the antibody moiety of a classical immunocytokine binds its cellular target, the ensuing increase of cytokine-receptor avidity translates into a modest increase of cytokine potency. **(C,D)** By introducing a mutation that decreases the affinity of the cytokine for its receptor, the activity of the mutated immunocytokine is now focused with a very high efficiency on target cells.

Since the late 70s the clinical potential of IFN-I for the treatment of patients suffering of viral infection or cancer diseases has been widely acknowledged (235). Today, this expectation is tempered because IFN-I treatment can induce severe side effects and sufficient doses cannot be administered in patients. Therefore, there is a strong need to create tuned IFN molecules devoid of side effects. Based on our current understanding of IFN-I responses as reviewed above, many parameters could be tuned individually or in a combined manner to modulate IFN-I activity to promote their beneficial effects over the deleterious ones in a number of diseases. These parameters include modifying the affinity of IFN-I for its receptor, playing with the local quantity/concentration of IFN-I and with the duration of its delivery, and modulating the nature of the cells that are responding to IFN-I. We will discuss here novel strategies being developed to deliver IFN-I to, or block IFN-I responsiveness of, a specific target cell type *in vivo* (Figure 10).

### Modulating IFN-I functions by altering their binding to their receptor through advanced biochemical engineering

If IFN-I-induced side effects are a consequence of the pleiotropic nature of IFN-I, and if the bioactivities mediating deleterious effects have some degree of independence from those mediating beneficial effects, one could mutate the IFN-I molecules in order to skew their activity toward a desired bioactivity. Indeed, introducing key mutation in IFN-α2 allowed increasing its affinity to IFNAR1 by a factor of 100. Accordingly, this IFN-α2 mutant is 100-times more potent in inhibiting cell proliferation, but as potent as WT IFN-α2 in inducing an anti-viral state (236–238). Hence, it is possible to tune IFN activity by modifying its binding to IFNAR. However, translating such an approach for the design of molecules for clinical application is severely hampered by the poor understanding we have on the IFN-I bioactivities mediating the side effects. Furthermore, we are far from having established the list of bioactivities that could be differentially modulated by changing the stability of the IFN-I/IFNAR complex. We know more about the cell types that mediate beneficial versus deleterious IFN responses in various diseases. Hence, we will now discuss strategies aimed at focusing IFN activity to specific cell types to promote health over disease.

### Cell specific targeting of exogenous IFN-I

Several strategies have been developed to specifically target IFNs on tumor cells, tumor-infiltrated immune cells or infected tissues. These strategies include intra-lesional injection (239, 240), adenoviral-mediated gene transfer (241–243), engineered tumor-infiltrating monocytes (244), and fusion of IFNs with a cleavable protecting shell (245). Another strategy to increase cytokine accumulation within the tumor or infected tissue is antibody-mediated targeting of cytokine delivery, where a cytokine moiety is fused to an antibody directed against a specific cell surface marker (Figure 10). The fusion molecule retains both antigen-binding and IFN-I bioactivities, and is enriched at the targeted site upon *in vivo* injection (246–249). When targeted to human CD20, IFN-I inhibited the proliferation of lymphoma cells engrafted in immunodeficient mice (250). An IFN-I targeted to a tumor antigen can also amplify the therapeutic effect of the antibody by acting on tumor-infiltrated DCs, thus increasing antigen cross-presentation and antitumor cytotoxic T cell responses (249). On non-targeted cells, the antibody conjugation negatively impacts IFN-I potency, but only modestly (18, 248, 251) (Figure 10A). Fusion molecules generally retain full IFN-I biological activity on the cells expressing the antibody target (Figure 10B). Hence, this difference only leads to a modest ratio between the IFN-I specific activity measured on target and non-target cells (Figure 10B). Such a targeting efficiency is definitely too low to reduce the toxic effect of IFN-I administration, because it will not specifically focus IFN-I activities on “beneficial cells” without stimulating “deleterious cells.”

The engineering of immuno-IFN-I must be improved to reach the very high targeting efficacy required to significantly diminish the treatment side effects. We recently reported an innovative strategy reaching this goal (18). It is based on the postulate that the antibody moiety of an immuno-IFN-I stabilizes the IFN-I/receptor-complex by avidity. It also takes into account the fact that the biological potency of an IFN-I is proportional to the stability of the IFN-I/receptor complex up to a certain threshold beyond which increasing the stability does not increase its potency (238, 252). IFN-α2 and IFN-β are used in most immuno-IFN-I studies. They have evolved to retain close to maximal potency. Hence, their targeting by an antibody that only provides a modest gain in terms of biological potency. However, it is expected that decreasing the affinity of the IFN-I for its receptor, by introducing a mutation, would increase the targeting effect of the antibody (Figures 10 **C,D**). This is indeed the case. Using an IFN-I with a single point mutation that dramatically decreases its affinity for IFNAR2 (Figure 10C) allows engineering immuno-IFNs that are up to 1000-fold more potent on cells expressing the antibody target (Figure 10D). The three log targeting efficiency of these novel types of immuno-IFNs is found for various activities measured *in vitro* or *in vivo* when delivered in mice. If the toxic side effect experienced by the patients treated with IFN-I is due to systemic IFN-I activity, this targeting technology may find considerable clinical applications since such engineered immuno-IFNs are virtually inactive while “en route” and are activated only after binding of the fused antibody to the desired target. It remains to define the useful targets according to pathologies, for example, tumor cells themselves and professional cross-presenting XCR1^+^ DCs for cancer (97, 98, 249), or hepatocytes for chronic HCV infection.

### Cell specific blockade of endogenous IFN-I

To treat autoimmune diseases, novel therapeutics targeting IFN-I have been developed, including two IFN-α-neutralizing monoclonal antibodies currently in clinical trials (Sifalimumab and Rontalizumab) (253, 254). However, long-term systemic neutralization of IFN-I activity may increase susceptibility to viral infection and tumor development. Alternative strategies are needed to specifically inhibit IFN-I deleterious effects in these diseases without globally compromising IFN-I anti-viral and anti-tumoral functions. The sequential nature of the assembling of the IFN-I/receptor complex opens the possibility to design IFN-I antagonists specifically targeting the cell subsets responsible for IFN-I deleterious effects.

An IFN-α2 carrying a single amino acid substitution that blocks the IFN-I/IFNAR1 interaction engages IFNAR2 in a complex, which cannot bind IFNAR1 (255). Since the binary IFN-I/IFNAR2 complex is devoid of any IFN-I activity, such mutant behaves as a potent IFN-I antagonist. When linked to an antibody specific for a cell surface marker, the antagonistic activity of the mutant IFN-I should be significantly reinforced specifically on the cells expressing the target. Hence, it should be possible to design and construct targeted antagonists that inhibit responsiveness to endogenous IFN-I specifically on the cell subsets on which the cytokines act to promote autoimmunity or severe side effects, leaving the other cells fully responsive. For example, in chronic HCV patients treated with Peg-IFN-α, one of the most deleterious side effects is nervous depression, which might be prevented by co-administration of an IFN-I antagonist specifically targeting neurons or other cells of the central nervous system.

## Conclusion

In the last decade, several major technological breakthroughs and the generation of novel animal models have remarkably advanced our understanding of the mode of action of IFNs. *In vitro* high throughput screening allowed systematically studying the functions of ISGs by ectopic expression or knock-down. Advance biophysical investigation of the interactions between IFN-I and the IFN-I receptor allowed to rigorously investigate the mechanistic basis for the differential bioactivities of IFN-I subtypes. The analyses of the responses of different cell types to IFNs or to viral infection, *in vitro* but also *in vivo* in various pathologies, demonstrated that IFN-I often mediate beneficial versus deleterious roles by acting on different cell types. From integrative analysis of these data, a picture is now emerging suggesting that it will be possible to segregate protective from deleterious IFN-I effects, based (i) on their differential induction depending on IFN-I subsets or on the magnitude/timing of IFN-I production, (ii) on their conditioning in different tissues, (iii) or on their occurrence in different cell types. Hence, innovative immunotherapeutic treatments are being designed to tune IFN-I activity toward desired effects in order to promote health over disease in a manner adapted to each physiopathological condition. In particular, a proof-of-concept has been made *in vitro* that it will be possible to target IFN-I activity on given cell types or tissues to administer to patients sufficiently high doses of the cytokine at the site of interest while limiting unwanted effects in other tissues or cell types. The next steps will be to demonstrate efficacy of this strategy *in vivo* in preclinical animal models. Importantly, to foster the development of these innovative immunotherapies, major efforts are still warranted to continue delineating which cell types are mainly responsible for the protective versus deleterious effects of IFN-I in different diseases. Combining high throughput technologies and systems biology approaches will also advance our understanding of the molecular mechanisms dynamically controlling IFN-I responses in health and diseases, which should reveal potentially novel therapeutic targets.

## Conflict of Interest Statement

The authors declare that the research was conducted in the absence of any commercial or financial relationships that could be construed as a potential conflict of interest.
